# Modular Metamaterials for Adaptable MRI Signal Control: Combining Dipole Array With a New Hexagon‐Based Artificial Dielectrics

**DOI:** 10.1002/advs.77378

**Published:** 2026-08-29

**Authors:** Santosh Kumar Maurya, Ilan Goldberg, Rita Schmidt

**Affiliations:** ^1^ Department of Brain Sciences Weizmann Institute of Science Rehovot Israel; ^2^ The Azrieli National Institute for Human Brain Imaging and Research Weizmann Institute of Science Rehovot Israel; ^3^ Department of Neurology Rabin Medical Center Petah Tikva Israel

**Keywords:** artificial dielectric, magnetic and electric dipole, metamaterial, modular design, tunable array of dipoles, ultra‐high field MRI

## Abstract

Emerging concepts of metamaterials for MRI offer improved image quality through enhanced RF field control. However, current designs are often limited by bulky configurations, dielectric losses, and limited adaptability. Patient‐specific optimization—essential for improving field homogeneity, boosting local SNR, and addressing anatomical variability—requires redesigning each setup. To overcome these limitations, we introduce a hexagonally‐packed artificial dielectric, free of any lumped‐elements, capable of achieving a wide range of high relative permittivities (ε_r_ ≈ 30–1400) suitable for clinical and ultra‐high‐field MRI. We identify multilayered and in‐plane‐shifted hexagonal configurations that support compact, modular layouts. Furthermore, we combine these artificial dielectrics with passive‐dipole array to create a modular system for adaptable signal enhancement, tuned via electric and magnetic dipole modes by adjusting the length of conductive strips. In vivo 7T MRI results demonstrate enhanced field control and high‐resolution imaging. This lightweight, flexible, and modular platform enables patient‐specific RF field shaping, thus opening up new avenues for personalized and adaptable MRI technology.

## Graphical abstract

A flexible, hexagonally packed artificial dielectric providing a wide range of effective permittivities (εr ≈ 30–1400) without lumped elements was developed for clinical and ultra‐high‐field MRI. Combined with modular passive‐dipole arrays, the system enables adaptable RF field shaping through electric‐ and magnetic‐dipole tuning. In vivo 7T MRI demonstrates improved image quality, establishing an anatomy‐adaptive platform for enhanced MRI performance.

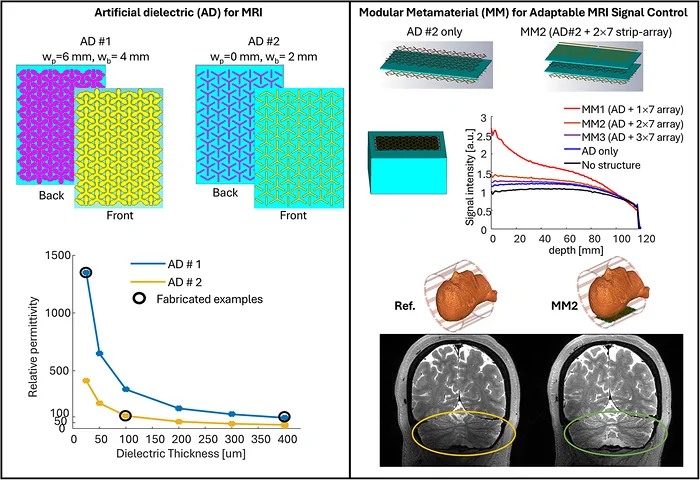

## 1 Introduction

Magnetic resonance imaging (MRI) has become a cornerstone of modern medical diagnostics, with over hundreds of million examinations conducted annually worldwide. Advances in parallel imaging using multi‐channel receive coils and the development of ultra‐high‐field MRI systems (≥7 Tesla) have enabled sub‐millimeter, 3D imaging of the human brain [1], further advancing the promise of personalized medicine. Yet, MRI is an approach inherently limited by its signal‐to‐noise ratio (SNR), always in need of higher SNR to enable higher spatial resolution and shorten scan duration. Shorter scan durations are paramount for both patient comfort and better image quality, as they offer greater resilience to motion artifacts.

Higher SNR is typically achieved by placing the receive coils as close as possible to the patient, thereby maximizing the filling factor. However, standard coil arrays are designed with fixed geometries and patient comfort in mind, leading to suboptimal configurations for individual anatomies. Recent wireless technologies [2, 3, 4, 5, 6, 7]—including dielectric pads and metamaterials—have demonstrated localized SNR improvements in brain, body, and functional MRI.

In addition to reception, metamaterials concepts can contribute to improving transmission [8, 9, 10]. MRI relies on spin‐excitation induced by the radiofrequency (RF) transmit field, commonly referred to as B_1_^+^, which determines the flip angle according to |γB_1_^+^τ|, where γ is the gyromagnetic ratio and τ is the RF pulse duration. Inhomogeneity in the B_1_^+^ field can lead to local signal loss and degraded diagnostic contrast. Such RF field inhomogeneity arises when the wavelength at the corresponding RF Larmor frequencies [11, 12, 13] becomes comparable to the dimensions of the scanned object —a challenge encountered during abdominal imaging at 3T and the imaging of any body part at ≥7T. Although much progress has been made to provide MR hardware solutions for this issue, especially multi‐channel transmit coils [14, 15, 16, 17, 18, 19, 20], flexible and local solutions can offer localized alternatives.

Dielectric materials added as pads to the MRI setup have previously been shown to improve RF homogeneity [21, 22, 23, 24], increase local SNR [25, 26, 27, 28, 29, 30], and reduce power deposition [21, 26, 31]. However, such setups are typically bulky and subject to significant dielectric losses. To address these limitations, metamaterials have emerged as promising alternatives [4, 32, 33, 34, 35, 36], offering compact and wireless means to improve RF field control. Recently, metamaterials composed of *artificial* dielectrics [37, 38, 39] have been shown to provide thinner and lighter solutions. Nevertheless, these designs rely on lumped capacitive elements, which increase fabrication complexity and contribute to additional losses. Despite recent progress, achieving high effective permittivity while maintaining thin and flexible artificial dielectric designs remains a significant challenge.

Dielectric and metamaterial structures in MRI can be designed either to enhance or suppress the RF magnetic field. Their electromagnetic response is primarily determined by the resonant frequency and dimensions of the structure. When the resonant frequency is above the Larmor frequency, the structure behaves predominantly as a dielectric loading element and provides moderate RF field enhancement. Examples include CaTiO_3_‐water dielectric pads [40] used at 7 T (relative permittivity ≈110, estimated resonance frequency 350–450 MHz) and BaTiO_3_‐water pads [41] used at 3 T (relative permittivity ≈300, estimated resonance frequency ≈170 MHz). When the resonant frequency approaches the Larmor frequency, induced currents and field localization are maximized, resulting in the strongest RF field enhancement. Examples include resonators developed for RF transmission in neck [42], calf [32, 43], and body imaging [44, 45]. In contrast, resonances below the Larmor frequency produce out‐of‐phase currents that suppress the local (B_1_^+^) field and can be exploited for local field suppression [46] and SAR reduction. Figure S1 illustrates these three operating regimes. Therefore, precise control of the resonant frequency is essential and motivates the development of tunable structures.

A major challenge of current metamaterial setups is that once fabricated, their electromagnetic (EM) properties are fixed. Patient‐specific optimization—essential for improving field homogeneity, boosting local SNR, and addressing anatomical variability—requires redesigning each setup, including capacitor values and structure dimensions. As a consequence, there is a growing demand for metamaterials capable of tailoring local SNR enhancement in a patient‐specific manner.

**FIGURE 1:**
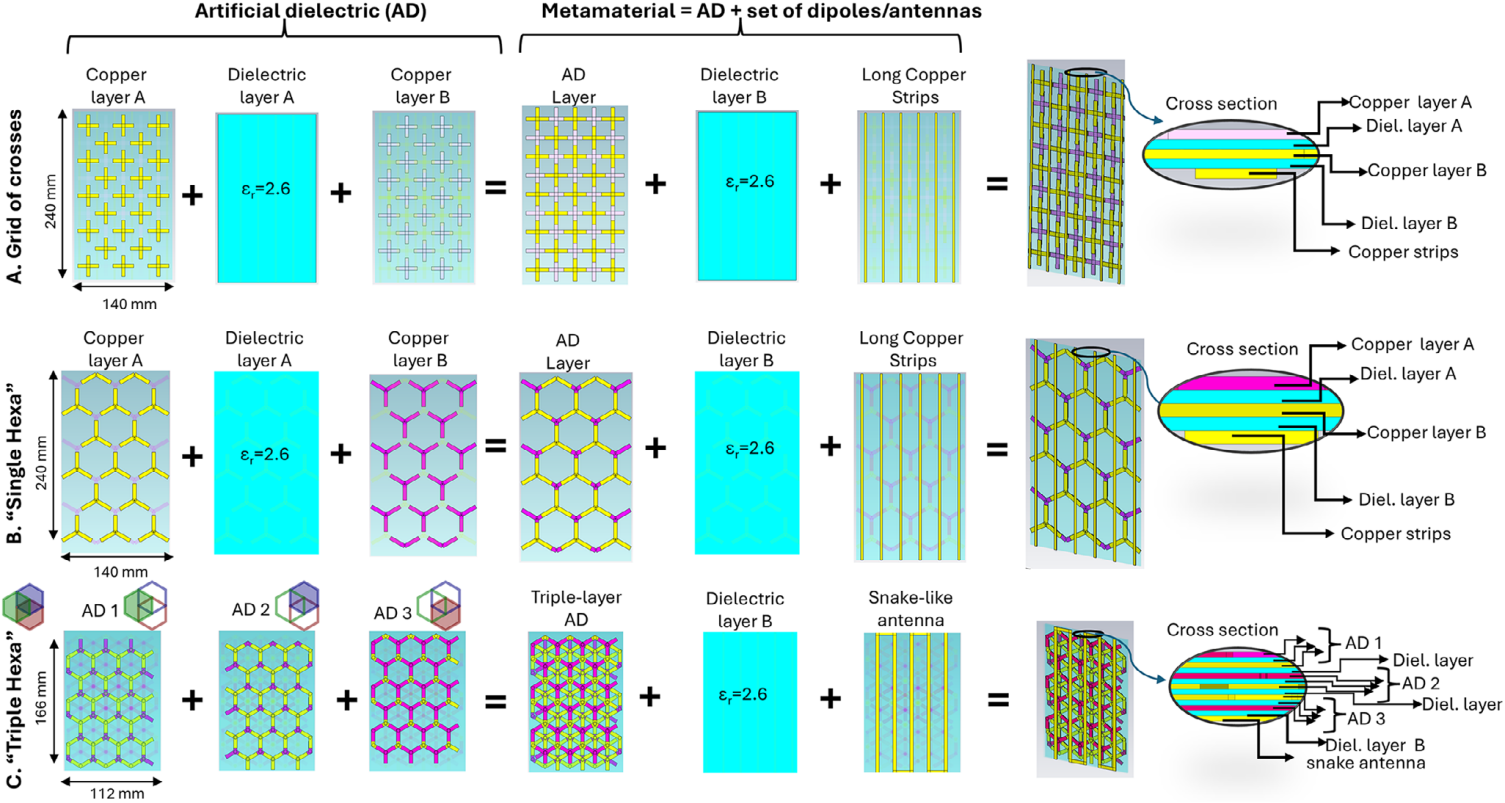
Schematic metamaterial design. Top to bottom shows (A) “Grid of crosses”, (B) “Single‐Hexa”, (C) “Triple‐Hexa” configurations. From left to right the schematics of the AD is shown and then the combined setup of the metamaterial. A cross section of the layers is included. The “Triple‐Hexa” AD includes three AD layers, where two layers are diagonally shifted and attached to the first one. The shift between the layers is depicted by the picture of three hexagons (green, blue and red).

In this study, we present two complementary strategies to address these challenges: achieving high effective permittivity in thin artificial dielectrics and enabling tunable electromagnetic responses for patient‐specific MRI applications. First, we develop an *artificial* dielectric free of lumped elements, based on a hexagonal lattice known for its high packing efficiency (a feature frequently exploited in natural systems). Such a lattice provides a higher packing density and a more isotropic distribution of neighboring elements than conventional square arrangements, which is advantageous for achieving a more homogeneous electromagnetic response. Our previous work demonstrated that a hexagonal lattice geometry can also reduce local electric‐field hotspots [32], which is particularly beneficial for MRI applications. Building on recent capacitive‐grid‐based dielectrics [37], we propose a structure comprising two shifted networks of conducting strips, on different sides of a dielectric substrate, and with each having sub‐wavelength gaps arranged in a hexagonal pattern (see Figure 1). The hexagonal pattern enables high relative permittivity suitable for clinical and ultra‐high field MRI applications. We further explore multilayer and in‐plane‐shifted configurations to enhance EM field interactions and support more compact layouts. These principles enable easy implementation of thin, flexible metamaterials, with the multi‐layer configurations offering a modular approach for tailored signal enhancement.

Second, we augment these artificial dielectrics with arrays of conducting strips to construct reconfigurable metamaterials. By varying strip lengths, we control electric and magnetic dipole resonances, enabling tunable signal enhancement in specific regions of interest. Our previous work [47] analyzed the contribution of conducting‐strip arrays supporting electric‐ and magnetic‐dipole‐like responses in a hybrid structure combining passive‐dipole arrays with a high permittivity dielectric material, demonstrating the ability to tailor the transmit RF magnetic field in MRI. Here, we combine the new flexible *artificial* dielectric with an array of copper strips of varying lengths and demonstrate the benefits of this hybrid structure for in vivo imaging. This design offers modularity by enabling easy switching between configurations, thus easily controlling electromagnetic properties and signal enhancement, paving the way for patient‐adaptable applications. We demonstrate its benefit in MRI applications relevant for clinical and research studies.

## 2 Results

### 2.1 Hexagonally Packed Artificial Dielectric—Single, Multi‐Layer and In‐Plane‐Shifted Configurations

To achieve compact subunit designs for artificial dielectrics (AD) suitable for MRI applications, we identified a hexagonal packing configuration capable of providing the desired range of relative permittivity. In this design (Figure 1B), six conductive three‐blade‐propeller shaped subunits are arranged to form a hexagon. Three subunits are laid on one side of the dielectric substrate, interleaved with three on the opposite side, creating a layered structure. A beehive‐like assembly of such hexagons forms a distributed capacitive network. For benchmarking, a conventional rectangular layout (Figure 1A)—similar to previously reported designs [37, 38, 39]—was assembled. The hexagonal arrangement enables the formation of an effective capacitor network without the need for discrete lumped components, thereby simplifying fabrication and enhancing the achievable effective permittivity.

#### 2.1.1 Single‐Layer Hexa

In this study, the hexagonal artificial dielectric (AD) was designed for brain imaging applications; therefore, the overall dimensions were selected to accommodate integration within a standard RF head coil setup. The configuration shown in Figure 1 was designed with a footprint of 24 × 14 cm^2^ accordingly. As a first step, a hexagonal layout was designed (Figure 1B) and compared to rectangular grid‐of‐crosses layout (Figure 1A). The hexagonal configuration achieved a relative permittivity of ε_r_ ≈ 80, a twofold increase compared to the conventional grid‐of‐crosses layout (ε_r_ ≈ 40) of the same overall dimensions. Note that the effective permittivity can be further optimized in both configurations. The effective permittivity values reported for the artificial dielectric above were based on simulations using 400‐micron‐thick dielectric substrate with ε_r_ = 2.6, selected to demonstrate feasibility with readily available materials—corresponding to the thickness of commonly available plastic sheets. This approach supports low‐cost prototyping and broader accessibility for practical implementations. Additionally, with flexible PCB printing technologies, substrates of thicknesses of 25, 50, 100, 200 microns can be utilized, enabling the fabrication of ultra‐thin and conformal artificial dielectrics. As the substrate thickness decreases, the effective permittivity increases. Reducing the substrate thickness to 25 µm results in a 16‐fold increase in relative permittivity compared with the 400‐µm‐thick configuration. This range of substrate thickness yields an overall relative permittivity values of 80 to 1280 for the hexagonal configuration. Different PCB materials can be also utilized to further increase effective permittivity.

#### 2.1.2 Multi‐Layered Hexa

Another configuration of interest is an artificial dielectric designed for smaller overall dimensions—for example, to achieve localized enhancement in the auditory region of the brain. To maintain the same relative permittivity as the larger design (ε_r_ ≈ 80) with a comparable number of subunits, we explored the potential of multilayered structures. A triple‐layered hexagonal design (Figure 1C) was configured with overall dimensions of 17 × 11 cm^2^ (with ∼two‐times smaller area than in Figure 1B), where each layer was laterally shifted to create overlapping regions, thereby increasing the overall capacitive network. This stacking approach yielded a 1.8‐fold increase in permittivity for a double‐layered setup, and a 2.5‐fold increase for the triple‐layered configuration, while maintaining the same footprint (Figure 1C). This compact multilayered design offers three key advantages: (1) higher permittivity within the same physical dimensions, (2) support for smaller layouts without compromising the operational frequency range, and (3) a modular architecture that allows the tuning of the overall permittivity based on patient‐specific anatomy and/or on application.

#### 2.1.3 Single Layer In‐Plane‐Shifted Hexa

**FIGURE 2:**
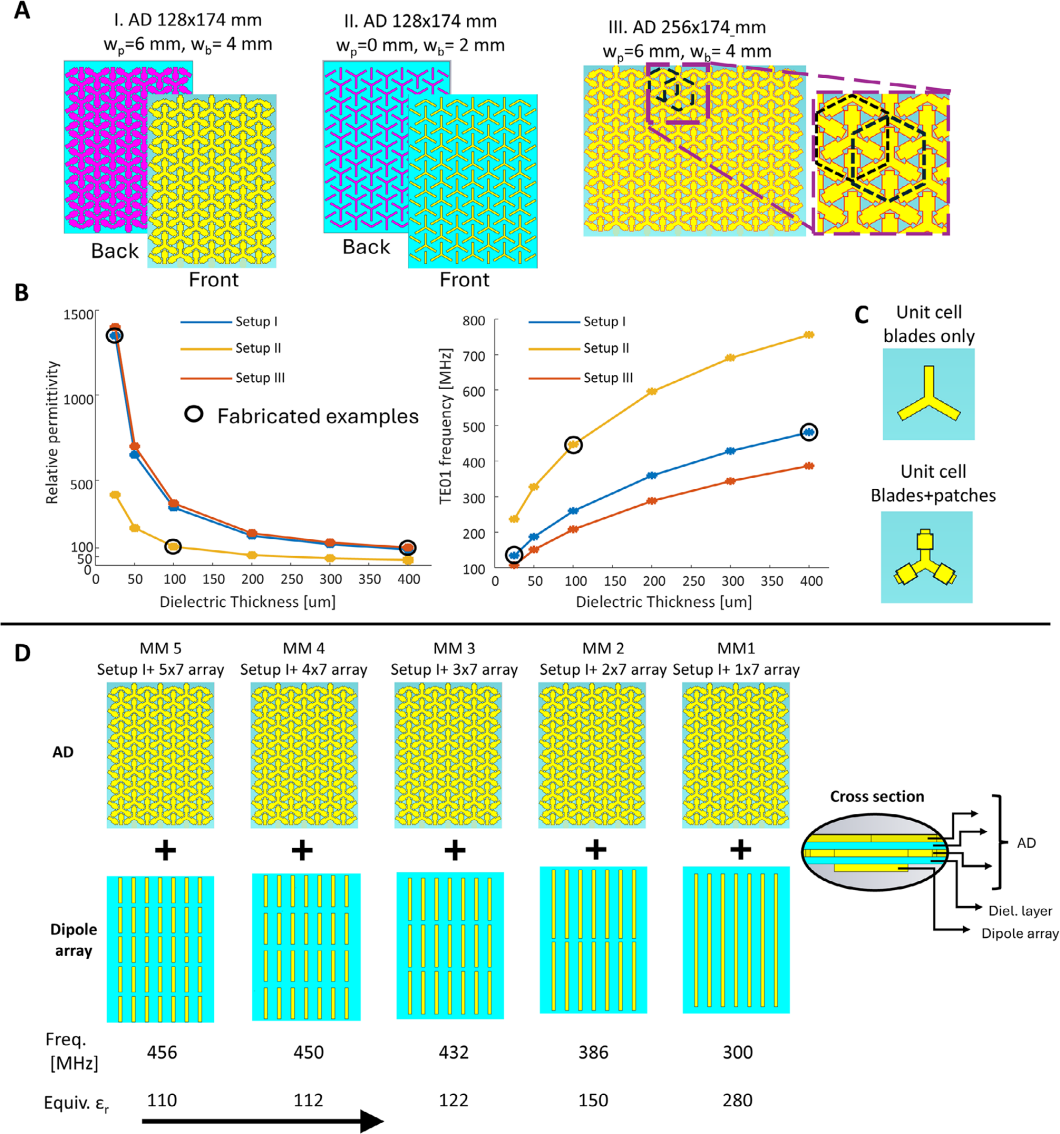
Combining artificial dielectric and dipole array. (A) In‐plane shifted hexagonal configurations. Simulations for three setups are shown: I) Layout with subunits containing blades and patches (using a 400‐micron dielectric substrate with ε_r_=2.6, yielding ε_r_ = 95); II) Layout with subunits containing blades only (using a 100‐micron dielectric substrate with ε_r_=2.6, yielding ε_r_ = 95); III) Setup with twice the in‐plane dimensions compared to I and II. (B) Relative permittivity (ε_r_) and TE_01_ mode frequency as a function of dielectric substrate thickness for the three layouts shown in A. (C) Schematics of a single unit cell with and without patches. (D) Modular setup combining the artificial dielectric with dipole array of varying lengths. Below each configuration, the corresponding TE_01_ frequency and equivalent ε_r_ (calculated for a homogeneous dielectric with the same frequency, in‐plane dimensions, and 7 mm thickness) are indicated.

Since multilayered designs increase the overall thickness, an alternative approach using in‐plane shifted hexagons was examined to further increase the effective permittivity (Figure 2). Here we successfully achieved ε_r_ ≈ 100 with 400‐microns thick dielectric substrate (Figure 2A, setup I), which is a value previously shown [29, 48] to be effective for brain applications using conventional dielectrics at 7T MRI.

To provide control over the resonance frequency and effective relative permittivity, the design incorporated the following parameters: blade width (w_b_), patch width (w_p_), and dielectric substrate thickness. Figure 2A (Setups I and II) illustrates configurations with and without the added patches, respectively. Additionally, the setup was tested with overall dimensions twice as large (Figure 2A, setup III), making it more suitable for body imaging applications. Considering the dielectric substrate thicknesses supported by flexible PCBs, the examined setups achieved relative permittivity values ranging from 30 to 1400 (Figure 2B). This broad range supports diverse applications—including brain [22, 28, 29, 49] and body imaging [31, 50, 51]—across MRI field strengths with relevant applications for 1.5T MRI and above, with a particular interest at 3T and 7T.

**FIGURE 3:**
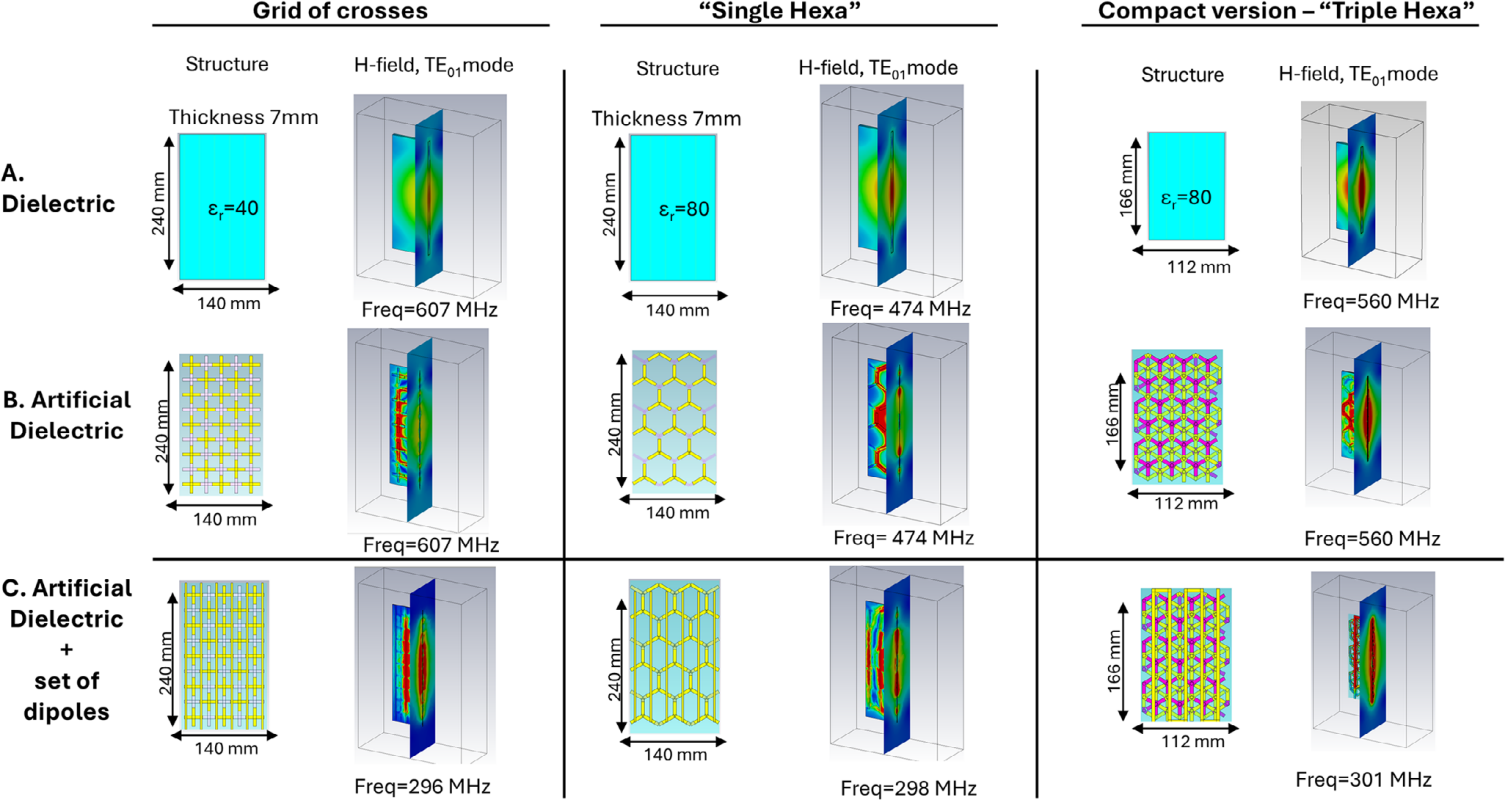
H‐field maps of AD and metamaterial configurations. The H‐field of the TE_01_ mode for each AD and the metamaterial configuration is shown. The AD H‐field is also compared to a 0.7 cm thick dielectric layer with the same resonant frequency. All three metamaterial configurations were designed to generate a TE_01_ resonance at ∼298 MHz. Each map was scaled separately to optimally visualize the distribution.

To compare the performance of artificial dielectrics to conventional dielectrics, we analyzed the lowest transverse electric (TE_01_) mode for each configuration. The effective permittivity of the artificial dielectric was defined as the relative permittivity of a 0.7 cm thick conventional dielectric, sharing the same in‐plane dimensions and TE_01_ resonant frequency (Figures 2 and 3). Figure S2 shows |H|‐ and |E|‐field maps in two orthogonal cross‐sections, demonstrating the similarity of the field distributions generated by the artificial and conventional dielectric structures. The comparison was made to a 0.7 cm thick conventional dielectric, since it is a commonly used size in MRI applications. The TE_01_ mode is particularly relevant for MRI applications, as it generates magnetic field (H‐field) components perpendicular to the main static magnetic field (B_0_) and exhibits a large penetration depth. Critically, this mode also minimizes the electric field within the subject or in regions external to the structure, thereby reducing the potential for unwanted power deposition. The distribution of the TE_01_ mode of the artificial and the conventional dielectrics exhibited similar distributions.

### 2.2 Combining Passive‐Dipole Array With Artificial Dielectrics for Adaptive Signal Control

While artificial dielectrics alone can offer a wide range of tunable EM properties, each configuration typically requires a dedicated printed layout. To address this, we investigated a hybrid approach that combines artificial dielectric structures with a passive‐dipole array, consisting of conducting strips. The incorporation of the strip array introduces additional control over the EM field distribution. Similar combinations with conventional dielectric materials have been previously investigated [4, 32, 35, 47], but here we extend this concept to artificial dielectrics and increase design flexibility.

For each configuration shown in Figure 1, an array of long conducting strips was added on top of the artificial dielectric, separated by a thin dielectric substrate. As an initial test, strip lengths were tuned to set the TE_01_ mode resonance frequency to the 7T Larmor frequency (298 MHz). The base artificial dielectric structure—without the strip array—was intentionally designed to be non‐resonant at 7T, providing a local B_1_^+^ enhancement in the range of 10–40%.

While an array of long conducting strips (≥16 cm) in the presence of an RF field behaves as an array of electric dipoles, an array of short conducting strips (e.g., 3 cm, where 3 cm ≪ λ, and λ≈12 cm in brain tissue at 7T MRI) exhibits a response analogous to that of an array of magnetic dipoles, functionally similar to cut split‐ring resonators (Ref. [52, 53]). This configuration also supports a TE_01_ resonant mode, although it appears at a higher frequency compared to the long‐strip setup. To explore the RF field control with this hybrid system, we combined the AD structure shown in Figure 2A setup I with arrays of conducting strips of varying lengths, ranging from 3 to 16 cm (Figure 2D). The longest strips (16 cm) correspond to configurations dominated by electric dipole behavior, while the shortest strips (3 cm) exhibit characteristics of magnetic dipoles. These distinct electromagnetic responses are reflected in the surface current distributions along the strips (see Figure S3). Intermediate lengths provide a gradual transition between these two regimes, enabling continuous control over the EM response of the system. Figure 2D demonstrates these effects, showing that substantial control over the effective EM properties—and, consequently, the local RF field enhancement—can be achieved by modifying only the conducting strip array. All this while retaining the same underlying artificial dielectric structure, a structure which is more complex and time‐consuming to fabricate than simple strips. The resulting TE_01_ resonant mode of the hybrid structure provides similar distribution of the H‐field in all configurations (Figures S2 and S4). Table 1 summarizes the frequency values of the TE_01_ resonant mode for each combined configuration as well as for separate configurations of the artificial dielectric and the array of conducting strips. The addition of the artificial dielectric reduced the resonant frequency by approximately a factor of 2.7 relative to the strip‐array‐only configuration, bringing the resonance into the frequency range relevant for MRI applications. The strip arrays with varying strip lengths further provide a tuning range of approximately 200 MHz (300–482 MHz, including the configuration without strips).

**TABLE 1:** TE_01_ resonant frequencies of the artificial dielectric (AD), strip arrays, and the corresponding combined configurations presented in Figure 2D.

Configuration	AD+5x7 strips	AD+4x7 strips	AD+3x7 strips	AD+2x7 strips	AD+1x7 strips
Freq. of combined setup [MHz]	456	450	432	386	300
Freq. of strips‐array only [MHz]	1257	1248	1180	1125	820
Freq. of AD only [MHz]	482	482	482	482	482

By implementing the two components separately—the artificial dielectric and a set of interchangeable conducting strip arrays—the system can easily be reconfigured and adapted to suit specific applications, anatomical targets, or field strength requirements without the need for reprinting the entire structure.

To evaluate the performance of the above metamaterial configurations with varying strip lengths, experimental measurements with a rectangular phantom at a 7T MRI were performed. The results demonstrate that the maximum local B_1_^+^ enhancement varied depending on the chosen configuration, reaching 10%, 14%, 22%, and 70% for setups AD1, MM3, MM2, and MM1, respectively (Figure S5). A subset of these configurations (AD1 and MM2) was further tested using a 3D head‐shaped phantom [54] at 7T to better mimic realistic imaging conditions, yielding results consistent with those observed in the rectangular phantom experiments (Figures S6 and S9). To confirm that the observed enhancement does not arise merely from the presence of a conductive layer, but rather from the engineered electromagnetic properties of the proposed artificial dielectric, we performed additional measurements and simulations comparing the artificial dielectric with a solid metal plate. The results demonstrate a clear distinction between the two structures: the artificial dielectric produces substantial RF field enhancement, whereas the metal plate results in signal degradation (Figures S7 and S8).

Additionally, scans at a 3T MRI were performed using the MM1 configuration (Figure S10), as it features ε_r_ ≈ 300—a value previously shown [50] to be effective in brain imaging at 3T. In this case, a 50% increase of the maximum local B_1_^+^ was observed, confirming the adaptability and effectiveness of the proposed design across different magnetic field strengths.

**FIGURE 4:**
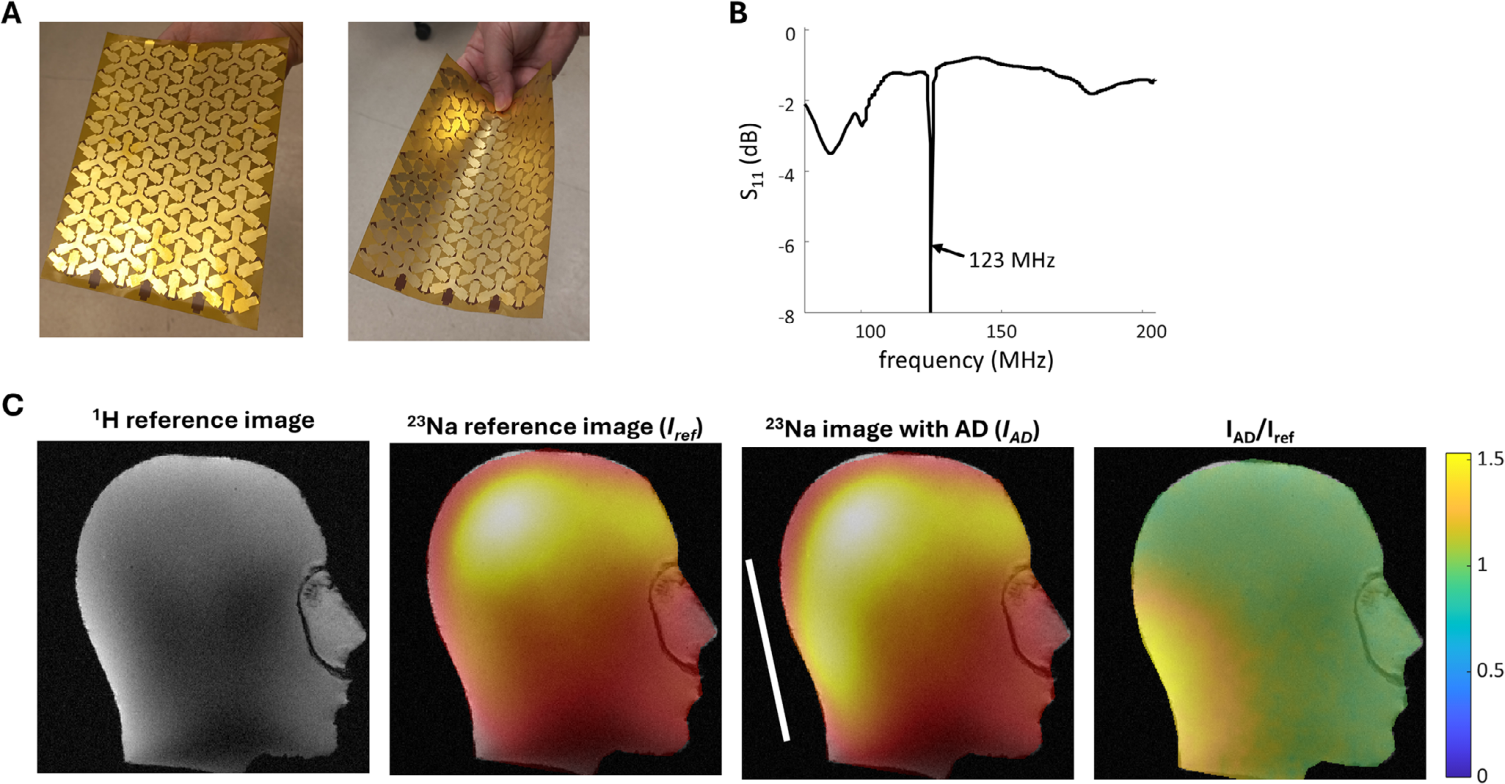
Artificial dielectric with ε_r_ ≈ 1250 fabricated on a 25‐µm dielectric substrate (total thickness: 97 µm). (A) Photographs of the experimental setup and the bending capability of the fabricated structure; (B) Measured frequency response (S11); (C) Sodium MRI (Larmor frequency ≈ 78 MHz) of a 3D head‐shaped phantom. From left to right: reference ^1^H image, reference ^23^Na image, ^23^Na image acquired in the presence of the artificial dielectric (AD), and the corresponding ratio map. The ^23^Na images are overlaid on the anatomical ^1^H image.

To further validate the proposed concept and assess its feasibility using thin PCB fabrication, we implemented a structure based on the Setup I configuration (Figure 2), consisting of a 25 µm dielectric substrate with 36 µm copper layers on both sides. Electromagnetic simulations demonstrated RF field enhancement comparable to that of a conventional dielectric with ε_r_ = 1250 (Figure S11). Experimental characterization confirmed the predicted behavior, with the measured resonance frequency (123 MHz) closely matching the simulated value (117 MHz) (Figure 4B). The structure was evaluated using sodium MRI at 7 T (Larmor frequency ≈78 MHz) (Figure 4). Experiments performed on a head‐shaped phantom demonstrated a 1.6‐fold increase in image intensity, corresponding to an estimated 1.26‐fold enhancement in B_1_^+^ (Figure 4C).

### 2.3 Adaptive Metamaterials Configurations for In Vivo Applications

**FIGURE 5:**
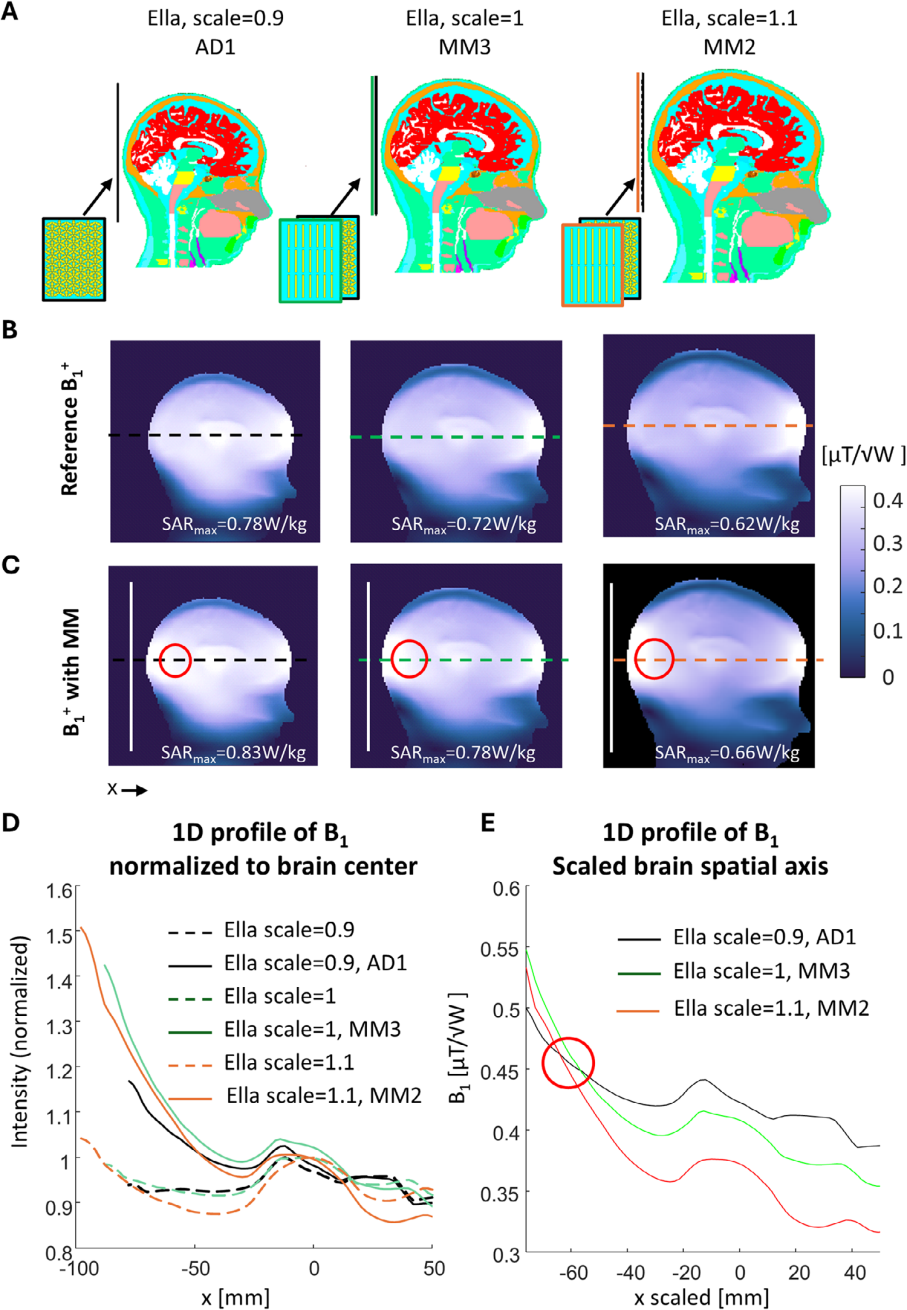
Electromagnetic (EM) simulations showing the effect of varying head size and of the metamaterial configurations designed to provide similar B_1_^+^ enhancement. (A) Simulation setup, (B) Reference B_1_^+^ maps in the central sagittal plane without the metamaterial, (C) B_1_^+^ maps with the metamaterial in place (the metamaterial location is shown as a white overlay). (D) 1D B_1_^+^ profile along the line indicated on the maps for each head size. (E) 1D B_1_^+^ profile along the same line as in D, with x‐axis scaled (x_scaled_) to the smallest head. The red overlay circle around x_scaled_=62 mm highlights the region corresponding to the visual cortex, where similar B_1_^+^ values were achieved across all three head sizes. Specific absorption rate (SAR) values for each setup are displayed on the corresponding B_1_^+^ maps.

Several brain regions—including the occipital lobe, cerebellum, and brainstem—are particularly susceptible to local signal dropouts in 7T MRI due to RF field inhomogeneities. Head size plays a significant role in these effects: larger head dimensions intensify destructive interference patterns, resulting in more pronounced signal loss, particularly in the posterior and temporal lobes. To investigate this phenomenon, EM simulations were conducted using the virtual human model “Ella” [55], with head dimensions scaled to 0.9×, 1.0×, and 1.1× of the original size. Simulations show that in the larger head model (1.1×), the maximum B_1_^+^ at the center of the head is 17% lower compared to the smaller head (0.9×), while the minimum B_1_^+^ within the brain in the displayed slice is reduced by approximately a factor of five (Figure 5).

To enhance B_1_^+^ intensity in the posterior brain region (containing the visual cortex) metamaterial configurations (artificial dielectric alone and MM1–MM4) were evaluated for their ability to provide local enhancement when placed as a thin pad at the back of the head. While phantom results showed that different metamaterial setups produced varying levels of B_1_^+^ enhancement, in this EM simulation set, we examined which configurations would yield similar B_1_^+^ distributions across different head sizes. Specifically, the AD1 only, MM3, and MM2 configurations with, respectively, the small (0.9×), medium (1.0×), and large (1.1×) head models, resulted in a similar normalized B_1_^+^ profile (Figure 5D). Table 2 shows the maximal specific absorption rate (*SAR_max_*) values and B_1_^+^ values at points of interest.

**TABLE 2:** Maximum B_1_^+^ enhancement for metamaterial setups shown in Figure 4 across varying head sizes.

	Ella, scale=0.9, AD1	Ella, scale=1.0, MM3	Ella, scale=1.1, MM2
Ratio of B_1max,_ with and without MM. Each profile was normalized to the center of the brain	1.25	1.46	1.44
Ratio of B_1_ with and without MM at x = −76 cm from the head center (corresponding to brain edge in Ella model scaled to 0.9). Each profile was normalized to the center of the brain.	1.24	1.32	1.30
B_1_ with MM at x_scaled_ = ‐62 mm (visual cortex area) [µT/√W]. x_scaled_ is a scaled axis matching the smallest head.	0.46	0.47	0.46
Ratio of B_1max_∕√SAR_max_ with and without MM. Each B_1max_ was normalized to the center of the brain.	1.23	1.35	1.34

An important objective in MRI is achieving the same spin flip angles within the imaging region of interest across different patients. In this example, scaling the resulting B_1_^+^ profiles of the three configurations to the same size (Figure 5E), we achieved similar B_1_^+^ amplitudes—and thus consistent flip angles—at the location of the visual cortex. For simplicity, we assumed that the position of a given brain area scales proportionally with head size. With the modular design introduced here, alternative metamaterial configurations can be selected and optimized for different anatomical targets or specific MRI applications.

### 2.4 Human Imaging at 7T MRI

**FIGURE 6:**
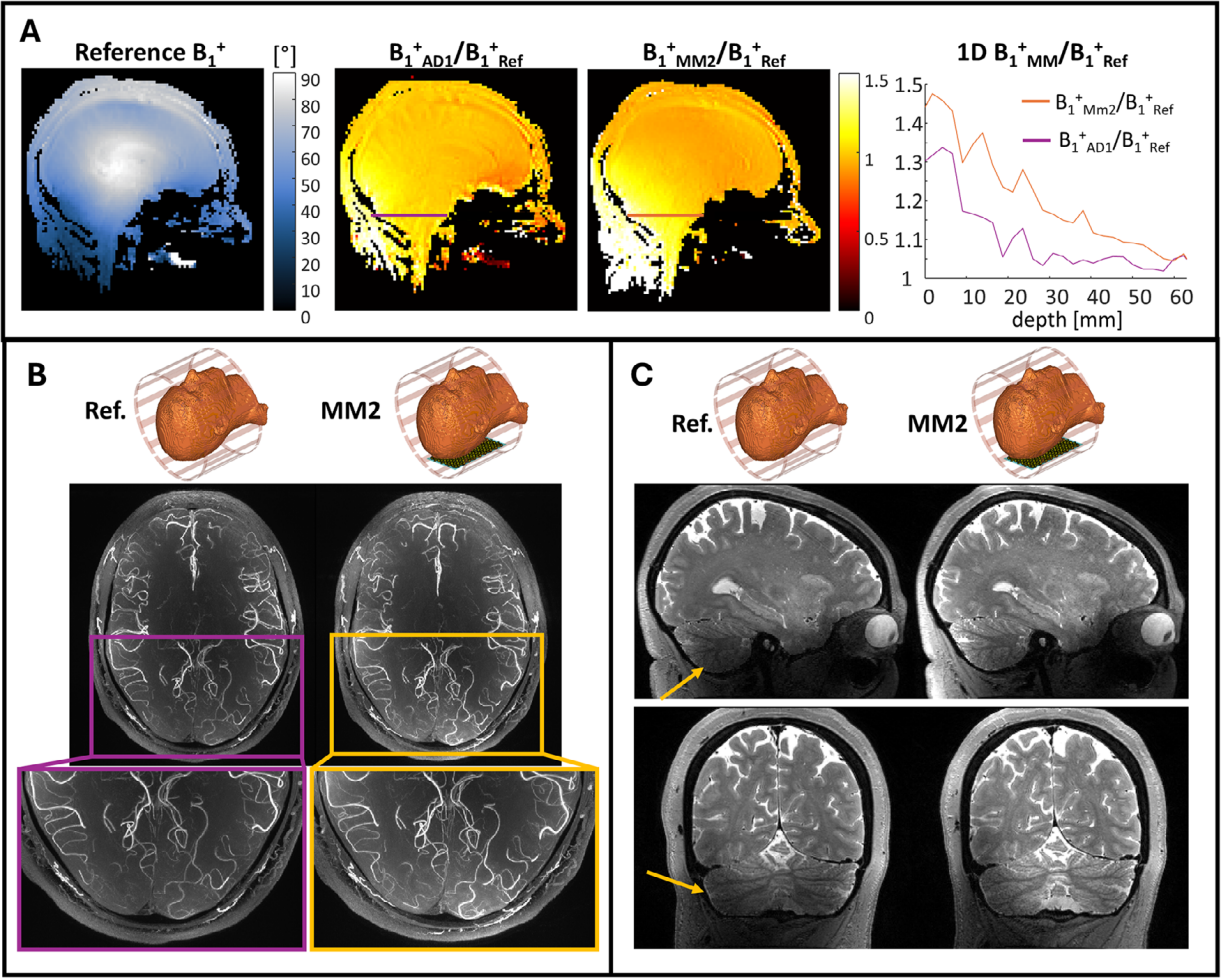
In vivo imaging demonstrating B_1_^+^ enhancement and improved image quality in posterior brain regions using an added metamaterial (A) Reference B_1_^+^ map and B_1_^+^ ratio maps with two metamaterial setups (AD1 and MM2), along with a 1D profile of the B_1_^+^ ratio along the line indicated on the maps. (B) Time‐of‐flight angiography maximum intensity projection (MIP) images without and with the MM2 setup. (C) Sagittal T_2_‐weighted images without and with the MM2 setup. The orange arrow highlights the cerebellum, where signal is markedly low without the metamaterial and significantly improved with the MM2 configuration.

To demonstrate the potential for local signal enhancement in vivo, B_1_^+^ field maps were acquired at 7T MRI using two metamaterial configurations (AD1 and MM2), resulting in localized B_1_^+^ increases of approximately 20% and 50%, respectively (Figure 6A). To further assess the impact of the enhanced transmit field across clinically relevant applications, three MRI sequences were acquired with and without the MM2 configuration. These included: (1) a non‐contrast‐enhanced time‐of‐flight (TOF) sequence for magnetic resonance angiography (MRA), used to visualize cerebral vasculature; (2) a high‐resolution 3D T_2_‐weighted sequence for detecting structural brain pathology; and (3) a multi‐echo gradient‐echo acquisition for generating quantitative T_2_* relaxation time maps, which serve as sensitive markers of microstructural integrity and long‐term pathological changes.

Angiography images acquired with the metamaterial configuration showed a pronounced improvement in vessel visualization in the posterior brain region, with a twofold signal increase in large vessels (Figure 6B). Some of the smaller vessels were below the SNR threshold without the added structure but became clearly visible when scanned with the metamaterial in place. T_2_‐weighted scans, which are particularly sensitive to RF field inhomogeneity due to the use of refocusing pulses (with signal intensity scaling approximately as B_1_^2^), also demonstrated substantial improvement. Specifically, signal intensity in the cerebellum increased by approximately 2.5‐fold (Figure 6C).

**FIGURE 7:**
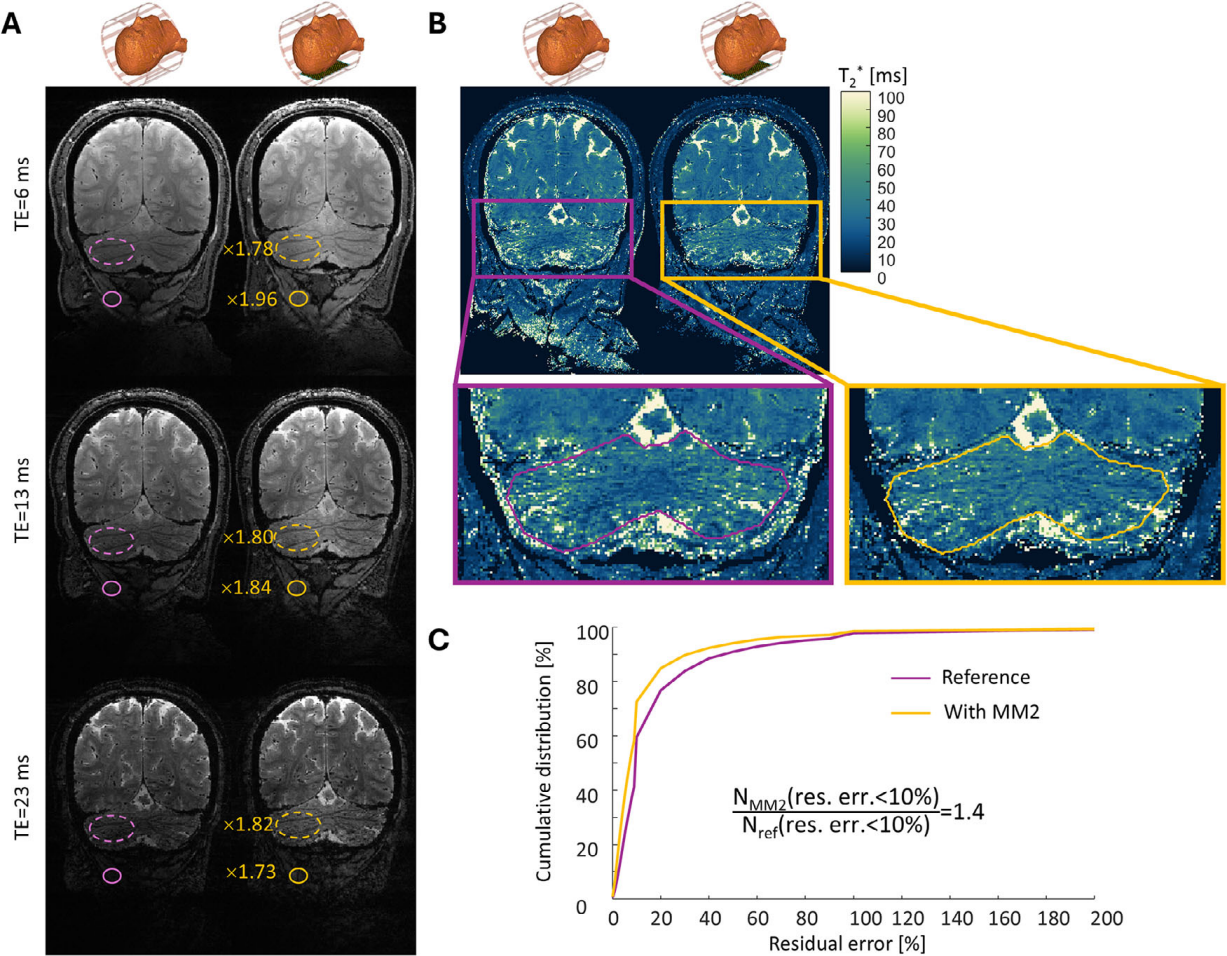
Improved quantitative T_2_* mapping performance in posterior brain regions using an added metamaterial. (A) Coronal magnitude images across the cerebellum acquired without and with the MM2 setup, shown for three echo times. Signal enhancement is highlighted in two regions of interest. (B) Corresponding T_2_* maps for the same slice, illustrating reduced overestimation artifacts in the cerebellar folds when using the metamaterial. (C) Cumulative distribution of residual errors in T_2_* estimation, comparing results without and with MM2.

Similarly, the multi‐echo gradient echo acquisition—used to sample signal decay over a range of echo times—showed a clear signal enhancement of 1.5‐ to 2‐fold in the cerebellum, particularly at longer echo times where signal levels are typically low (Figure 7A). Since quantitative T_2_* mapping relies on fitting the signal decay curve, low SNR in specific regions often leads to overestimation of T_2_* values. By improving local signal strength, the added metamaterial enhances the reliability of the quantitative mapping (Figure 7B,C). A cumulative distribution plot of relative fitting errors in the cerebellum revealed a 1.4‐fold increase in the number of voxels with error below 10%, confirming improved accuracy in T_2_* estimation.

## 3 Discussion

In this study, we developed and demonstrated a new class of modular MRI‐beneficial metamaterials combining hexagonally packed artificial dielectrics with passive‐dipole array. This modular platform enables local signal enhancement in MRI by tailoring the EM field in a flexible and adaptable manner.

One key advantage of the proposed artificial dielectric is its lumped‐ element‐free design, which leverages a hexagonal lattice to create distributed capacitive networks and achieve a wide range of effective relative permitivities (ε_r_ ≈ 30–1400), suitable for local signal enhancement from clinical to ultra‐high field MRI (1.5T‐7T and beyond). Through multilayered and in‐plane‐shifted configurations, we achieved fine control over permittivity, while maintaining compact and thin layouts, making the design practical for placement between the patient and a close‐fitting receive coil array. Compared to previously proposed rectangular geometries, the hexagonal configuration provided a substantial increase in effective permittivity, achieving up to a twofold enhancement in single‐layer implementations. Additional gains of up to 2.5‐fold were observed with multilayer stacking. The in‐plane shifted configuration was implemented using readily available materials—a 400‐micron plastic sheet and 4 mm‐wide copper tape—offering a low‐cost and easy fabrication approach. Additionally, flexible printed versions were fabricated using 25‐µm and 100‐µm dielectric substrates, demonstrating the compatibility of the proposed design with high‐precision fabrication processes. The selected substrate provided high mechanical reliability, with a manufacturer‐specified elongation at break of 50%. Both fabricated structures could be readily bent (Figure 4) and positioned beneath the head without mechanical damage. Nevertheless, applications requiring full conformity to complex anatomical surfaces may benefit from softer substrates, such as silicone‐based materials. Alternatively, improved conformity could be achieved through modular design composed of interconnected subunits, enabling adaption to complex geometries while maintaining mechanical robustness and electromagnetic performance.

Beyond control of the permittivity, the supplementing of dipole array adds a practical option of modularity. By varying the length of the strips, we demonstrated control over the resonant mode (TE_01_), shifting the frequency from 300 to 487 MHz, which in turn modulates the level of local B_1_^+^ enhancement. This tuning, achieved by simply interchanging layouts of the strip array while keeping the artificial dielectric base fixed, allows for field‐specific and anatomy‐specific adjustments without redesigning the entire structure. This modularity paves the way for reconfigurable designs tailored to patient‐specific clinical applications.

Our simulations and phantom measurements confirmed substantial local B_1_^+^ enhancement—up to 70%—depending on the configuration. We further validated the performance using a 3D head‐shaped phantom designed to mimic brain tissues at 7T and demonstrated cross‐field applicability with a configuration for 3T.

To address anatomical variability, our simulations highlight the importance of selecting configurations tailored to individual head size. We showed that B_1_^+^ amplitude decreases with increasing head size, and that achieving consistent excitation flip angles across different anatomies requires adaptable tuning of the metamaterial configuration. As a representative application, we demonstrated the mitigation of posterior signal dropouts—a known challenge in ultra‐high‐field brain imaging —by optimizing the configuration as a function of head size.

In this work, we demonstrated applications where local signal enhancement is particularly beneficial. While MRI signal intensity depends on multiple factors—including tissue properties and pulse sequence parameters—a useful approximation can be made in the low flip angle regime to estimate SNR behavior. Under these conditions, the image SNR is approximately proportional to sin(γB_1_^+^τ) · (B_1_^−^ *) / √P*, where *γ* is the gyromagnetic ratio*, τ* is the RF pulse duration*, P* is the accepted power of the coil, and *B_1_^−^* is the complex conjugate of the transmit field, defined as (B_1x_ − iB_1_ᵧ)/2. For small flip angles, this expression simplifies to SNR ∝ γ(B_1_^+^)^2^τ / √P, indicating that enhancements in both transmit (B_1_^+^) and receive (B_1_^−^) fields can contribute multiplicatively to the SNR. In regions affected by local B_1_^+^ dropout, improvements in B_1_^+^ may therefore yield an approximately quadratic increase in SNR—assuming a corresponding gain in B_1_^−^. However, this assumption should be applied with caution, as B_1_^+^ and B_1_^−^ serve distinct roles in excitation and signal reception, respectively, and can exhibit spatial differences. Their relative contributions depend on the specific MRI pulse sequence and acquisition strategy used. In our experiments, the observed increases in B_1_^+^ and image intensity—particularly in gradient‐echo acquisitions—suggest that this relationship held for the tested configurations. These findings indicate that both RF transmission (B_1_^+^) and reception (B_1_^−^) can be locally enhanced, resulting in improved SNR without the need for active circuitry or bulky hardware. While passive setups for local enhanced SNR were previously demonstrated with dielectric pads, the new design highlights new practical aspects—light, thin and modular—for RF field shaping.

Human scans at 7T MRI further support the practical utility of our approach. B_1_^+^ mapping showed 20–50% signal enhancement in targeted brain regions, and clinical sequences demonstrated the impact of this enhancement. Angiography revealed up to twofold signal increase in large posterior vessels, while T_2_‐weighted imaging showed clear improvement in the cerebellum—critical for assessing pathologies such as multiple system atrophy and posterior circulation stroke. The multi‐echo gradient echo scans, used for quantitative T_2_* mapping, benefited significantly from increased signal at longer echo times. This not only improved image quality but also enhanced the accuracy of T_2_* relaxation estimates. In the cerebellum, the reliability of T_2_* mapping improved significantly, with a 40% increase in the number of voxels exhibiting fitting errors below 10%.

Taken together, these findings underline the versatility, adaptability, and clinical relevance of the proposed metamaterial platform. The ability to easily reconfigure the system for different anatomical targets, field strengths, or clinical sequences, positions it as a compelling tool for personalized MRI. This solution beneficently offers low‐cost compact designs that easily integrate into existing workflows.

Future work may focus on expanding the platform to automatic reconfigurable implementation as well as imaging of other body regions—such as cardiac or abdominal imaging—where RF inhomogeneity and SAR constraints are pronounced. A reconfigurable implementation would enable independent control of the transmit and receive fields, which could be advantageous in applications where selective adjustment of either the flip angle distribution or the SNR is desired. Additionally, integration with data‐driven approaches (e.g., machine learning‐based B_1_^+^ mapping or real‐time feedback systems) could enable fully optimized, patient‐specific configurations at the point of care.

## 4 Materials and Methods

### 4.1 Characterization of the Metamaterial Structure

#### 4.1.1 Common Design Principle of the Artificial Dielectric

The basic characteristic of the AD is two laterally shifted copper strip grids separated by a dielectric substrate. In our simulations and experiments we used a dielectric substrate with a relative permittivity of ε_r_ = 2.6. This configuration forms a distributed capacitive network that determines the effective dielectric properties of the AD structure. The investigated AD designs featured hexagonal structures in single‐layer and multi‐layer (Figures 1 and 3), and in‐plane shifted (Figure 2) configurations.

#### 4.1.2 Single and Multi‐Layer AD Design and EM Simulations

In Figure 1 a hexagonally structured design (‘Single‐Hexa’) was compared to a conventional grid layout (‘Grid‐of‐Crosses’) to demonstrate more efficient spatial filling, resulting in a higher effective dielectric constant. A triple layer hexagonally structured design (‘Triple‐Hexa’) was designed to achieve the same permittivity as ‘Single‐Hexa’ but for smaller overall dimensions.

Electromagnetic (EM) simulations were performed using CST Studio Suite 2019 (Dassault Systèmes,  Velizy‐Villacoublay, France), including both eigenmode analysis (Figure 3) and full‐setup simulations. Due to the relatively small number of unit cells and the resulting finite‐size effects, conventional homogenization approaches are not directly applicable. Therefore, an eigenmode solver was used to characterize the AD structure, specifically its fundamental transverse‐electric (TE_01_) mode and corresponding resonant frequency. For each configuration, the effective ε_r_ was obtained by identifying the ε_r_ value of a conventional 0.7 cm thick dielectric yielding the same TE_01_ mode frequency. Additionally, the influence of the middle dielectric layer's thickness on the AD's effective permittivity was systematically evaluated (Figure 2B).

Single‐Hexa and Grid‐of‐Crosses configurations were each combined with a dipole array to form resonant structures at 298 MHz. Similarly, the Triple‐Hexa design was paired with a snake‐like antenna [56] to extend the effective electric dipole length, compensating for its shorter overall dimensions.

#### 4.1.3 In‐Plane Shifted AD Design and EM Simulations

Figure 2A illustrates AD structures based on in‐plane shifted hexagonal designs, demonstrated in two sizes: 24 × 14 cm^2^ and 48 × 14 cm^2^. The first two configurations incorporate subunit patches to tune the effective dielectric properties. The first was optimized for low‐cost fabrication, using a 400‐micron dielectric substrate and 4 mm copper strips. The second was designed for flexible PCB printing, employing 2 mm copper blades and a 100‐micron dielectric substrate. To achieve comparable effective permittivity with the thinner substrate, patches were included. For each configuration, the dependence of the effective relative permittivity and TE_01_ mode frequency on dielectric substrate thickness was calculated.

#### 4.1.4 Combining In‐Plane Shifted AD With Dipole Array

A modular design was developed by combining the AD setup #1 from Figure 2A with dipole array of varying lengths. Dipole lengths of 3, 3.6, 5, 8, and 15.6 cm were tested within a final layout of 17.4 × 12.8 cm^2^, implemented as arrays of 5×7, 4×7, 3×7, 2×7, and 1×7 dipole units, respectively (see Figure 2D). These configurations were referred to as MM5, MM4, MM3, MM2, and MM1, respectively. For each configuration, the corresponding TE_01_ resonance frequency and the enhancement of the RF transmit field were evaluated.

#### 4.1.5 Full‐Setup EM Simulations Details

Simulated B_1_^+^ maps were normalized to an accepted power of 1 Watt. Phantom simulations for estimating the B_1_^+^ field included a surface loop coil and a rectangular phantom mimicking brain tissue properties (ε_r_ = 53, σ = 0.3 S/m), with dimensions of 14 × 8 × 16 cm^3^. Human model simulations (Figure 4) incorporated a 8‐loop coil array designed to provide whole‐brain coverage (Figure S12). The overall coil coverage spanned over 30 cm in diameter (comparable to the Nova transmit coil) and 10 cm in length. To verify that similar enhancement can be achieved under realistic coil‐loading conditions, an additional set of simulations was performed using an 8‐channel coil with dimensions comparable to those of a commercial Nova head coil and a head‐sized cylindrical phantom. In this case, B_1_^+^ fields were compared for the baseline configuration, with the artificial dielectric, and with a metal plate replacing the artificial dielectric. The “Ella” human model from the Virtual Family was used with a mesh resolution of 1 × 1 × 1 mm^3^ to simulate the RF transmit field in the head region. To evaluate the impact of head size variability, the model's dimensions were scaled by factors of 0.9, 1.0, and 1.1. The metamaterial structure was positioned near the occipital lobe, as shown in Figure S4.

### 4.2 Metamaterials Implementation

Fabricated configurations included the Single‐Hexa, Triple‐Hexa (Figure 1), and three in‐plane shifted Hexa designs (two Figure 2I and one Figure 2II). Photographs of all setups are presented in Figure S13 and Figure 4. The setup shown in Figure 1 used a dielectric substrate with relative permittivity ε_r_ = 2.6, a thickness of 400 microns.

The flexible PCB versions of Figure 2 were fabricated by Newline‐PCB LTD (Haifa, Israel) using DuPont Pyralux AP—a double‐sided copper‐clad laminate with ε_r_ = 3.4. The design featured a 25‐µm and 100‐µm‐thick dielectric layers and 36‐micron copper layers printed on both sides according to our specifications.

### 4.3 MRI Measurements

MRI scanning was performed on a 7T system (MAGNETOM Terra, Siemens Healthcare, Erlangen) using a 1Tx/32Rx Nova coil, and on a 3T system (Prisma, Siemens Healthcare, Erlangen). Sodium MRI was performed using a dual‐frequency ^1^H/^23^Na head coil (1Tx/16Rx; Tesla Dynamic Coils B.V., The Netherlands). B_1_^+^ field maps were acquired using the vendor‐provided B_1_ mapping sequence, based on a preconditioning RF pulse with Turbo FLASH readout [57], with a 200 × 200 mm^2^ field of view (FOV) and spatial resolution of 2.5 × 2.5 × 3.5 mm^3^.

Phantom studies included: (i) a rectangular phantom composed of sucrose, agarose, and water, providing ε_r_ ≈ 57, σ = 0.3 S/m (mimicking brain tissue properties) (ii) a head‐shaped brain‐mimicking phantom as described in Ref. [54]; (iii) a single‐compartment head‐shaped phantom used for sodium MRI and (iv) the standard Siemens phantom—a cylindrical container filled with nickel sulfate hexahydrate and sodium chloride solution—used on the 3T system. In the head‐shaped phantom setup, the metamaterial was positioned at the back of the “head” to mimic a realistic in vivo scan. In all other setups, the metamaterial was placed on top of the phantom. Differences in image intensity in ^1^H MRI were assessed using a gradient‐echo (GRE) sequence. For sodium MRI, a vendor‐supplied ultra‐short echo time (UTE) sequence was used with 4‐mm isotropic resolution.

The in vivo study was approved by the Institutional Review Board of Rabin Medical Center (Petah Tikva, Israel; study number 0319‐25‐RMC), and all scans were performed after obtaining written informed consent.

The in vivo scans included B_1_^+^ maps acquired with two metamaterial configurations (AD setup 1 and MM2, shown in Figure 2), as well as a reference B_1_^+^ map without a metamaterial. Additionally, three MRI sequences were acquired using the MM2 configuration to assess clinically relevant applications. The parameters of these three scans are summarized below:

(1) A non‐contrast‐enhanced time‐of‐flight (TOF) sequence for magnetic resonance angiography (MRA)—FOV 220×176×33 mm^3^, spatial resolution 0.3×0.3×0.3 mm^3^, 2 slabs, bandwidth per pixel 120 Hz, TR/TE 27/5.61 ms, flip angle 15°, acceleration factor ×3, scan duration 6:44 min.
(2) A high‐resolution 3D T_2_‐weighted sequence for detecting structural brain pathology: SPACE sequence with FOV = 220 × 150 × 170 mm^3^, spatial resolution = 0.67 × 0.67 × 0.67 mm^3^, TR/TE = 4500/118 ms, acceleration factor ×6, scan duration 7:21 min.
(3) A multi‐echo multi‐slice gradient‐echo acquisition for generating quantitative T_2_* relaxation time maps: FOV = 210 × 240 × 12 cm^3^, spatial resolution = 0.75 × 0.75 × 0.8 mm^3^, acceleration factor ×2, TR = 2.8 s, five echo times TE = 6.44 ms, ΔTE = 3.3 ms, and flip angle = 55°.

## Author Contributions

**Santosh Kumar Maurya**: conceptualization, investigation, funding acquisition, writing – original draft, methodology, validation, visualization, writing – review and editing, software, formal analysis. **Rita Schmidt**: conceptualization, investigation, funding acquisition, writing – original draft, methodology, validation, visualization, writing – review and editing, software, formal analysis, project administration, data curation, supervision, resources. **Ilan Goldberg**: validation, writing – review and editing.

## Conflicts of Interest

The authors declare no conflicts of interest.

## Supporting information




**Supporting File**: advs77378‐sup‐0001‐SuppMat.pdf.

## Data Availability

The data that support the findings of this study are available From the corresponding author upon reasonable request.
