# Supplementary material for: Modular Metamaterials for Adaptable MRI Signal Control: Combining Dipole Array With a New Hexagon‐Based Artificial Dielectrics
Source: Adv Sci (Weinh). 2026 Aug 29:e77378. Online ahead of print. doi: 10.1002/advs.77378 (PMC13525418; doi:10.1002/advs.77378)
Supplement: Supplementary file 1 [file ADVS-9999-e77378-s001.pdf]

Supplementary Materials for

Modular Metamaterials for Adaptive MRI Signal Control: Combining Dipole Arrays with a New Hexagon-based Artificial Dielectrics

Santosh Kumar Maurya [1,2], Ilan Goldberg[3], Rita Schmidt[1,2]

[1]Department of Brain Sciences, Weizmann Institute of Science, Israel

[2]The Azrieli National Institute for Human Brain Imaging and Research, Weizmann Institute of Science

[3]Department of Neurology, Rabin Medical Center, Israel

This PDF file includes:

Figures S1 to S13

S1. Adding Dielectric Material: Three Operating Regimes

Dielectric and metamaterial structures in MRI can be designed either to enhance or suppress the RF magnetic field. Their electromagnetic response is primarily determined by the relationship between the structure's resonant frequency and the Larmor frequency. When the resonant frequency is above the Larmor frequency, the structure behaves predominantly as a dielectric loading element and provides moderate RF field enhancement. As the resonant frequency approaches the Larmor frequency, induced currents and field localization increase, resulting in the strongest RF field enhancement. In contrast, when the resonant frequency falls below the Larmor frequency, out-of-phase currents are induced, leading to local suppression of the transmit field (B_1^+). This regime can be exploited for applications requiring local field reduction or SAR mitigation.

Figure S1 illustrates simulated examples of these three operating regimes at 3 T. The simulations included a loop coil, a brain-mimicking phantom, and a dielectric structure with a thickness of 7 mm. The resonant frequency was varied by changing the effective relative permittivity of the dielectric. For one resonant frequency, two equivalent dielectric configurations are shown: one achieved by modifying the relative permittivity (ε_r=689 thickness=7 mm) and the other by adjusting the dielectric thickness (ε_r=300, thickness=18 mm), demonstrating that similar electromagnetic responses can be obtained through different design parameters.

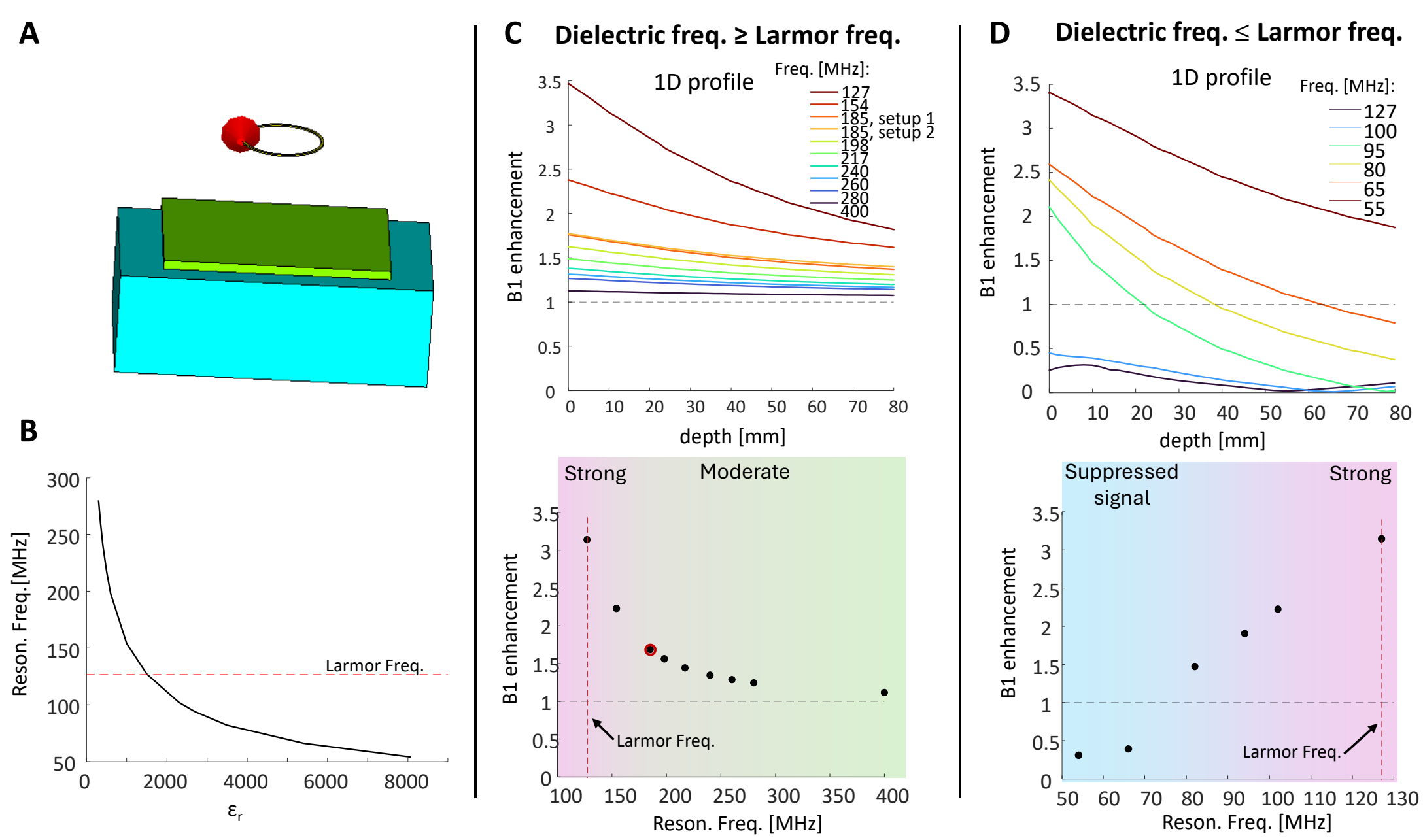

Figure S1. Adding dielectric material: three operating regimes. (A) Simulation setup. (B) TE_{01} resonant frequency of the dielectric as a function of relative permittivity for fixed dimensions of 128 × 174 × 7 mm³. 1D profiles of the normalized B_1 field (normalized to the case without dielectric) are shown for structures with resonant frequencies above the Larmor frequency (C) and below the Larmor frequency (D). The B_1 ratio measured 10 mm from the phantom surface is plotted for each case. Black symbols correspond to structures with varying relative permittivity, while the red circle denotes a structure with a different thickness. Cases exhibiting moderate enhancement are highlighted by the green region, near-resonant enhancement by the pink region, and signal suppression throughout the phantom by the light-blue region.

S2. TE resonant mode field and current distributions

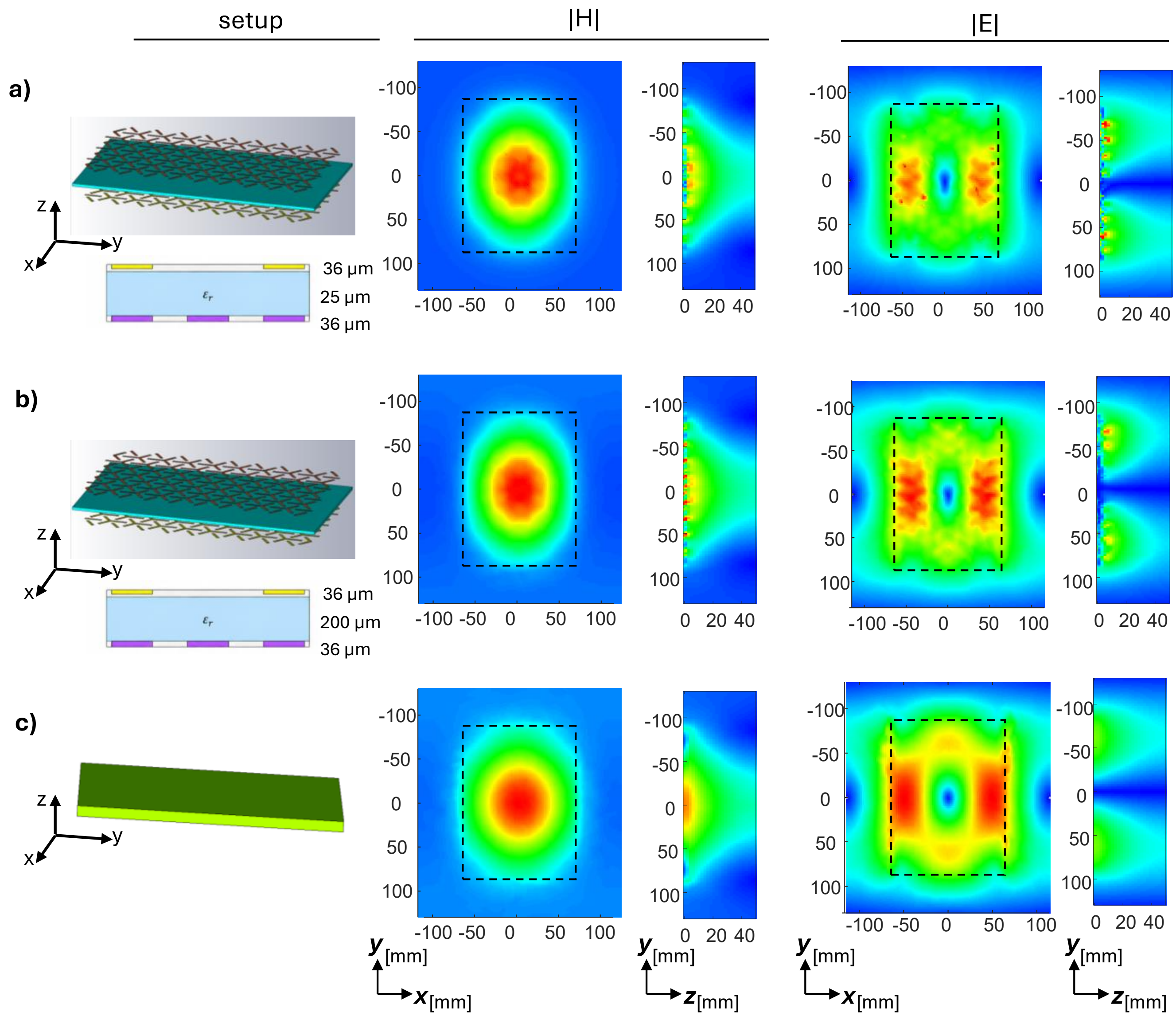

Figure S2. Resonant-mode magnetic and electric field distributions of artificial and conventional dielectrics. (A) Artificial dielectric with a 25-µm-thick dielectric substrate. (B) Artificial dielectric with a 200-µm-thick dielectric substrate. (C) Conventional dielectric. From left to right: structure schematics; |H| field maps in a plane parallel to the structure located 10 mm from its center and in a plane perpendicular to the structure; and |E| field maps in a plane parallel to the structure located 10 mm from its center and in a plane perpendicular to the structure. The black dashed outlines indicate the dimensions of the structures. No significant differences in the RF field distributions were observed between the artificial dielectrics with different substrate thicknesses or between the artificial and conventional dielectric structures.

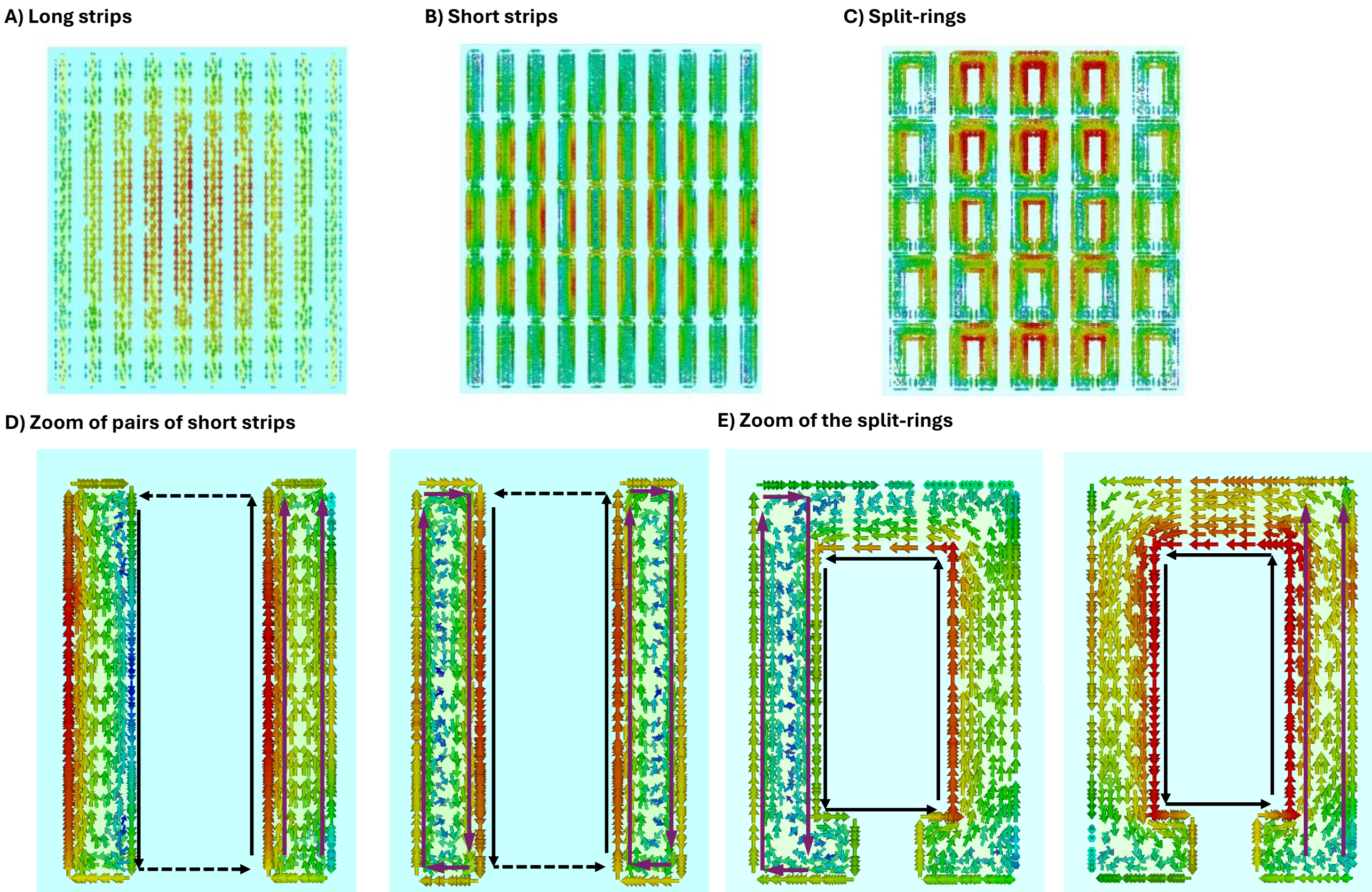

Figure S3. Surface current distributions for arrays of long strips, short strips, and split-ring resonators. Two pairs of short strips are enlarged and compared with two split-ring resonator units. In the long-strip array, the induced currents primarily flow along the strips from one end to the other without changing direction, characteristic of an electric-dipole response. In contrast, the currents in the short-strip array circulate between neighboring strips in a manner similar to that observed in split-ring resonators, forming loop-like current paths that generate an effective magnetic-dipole response.

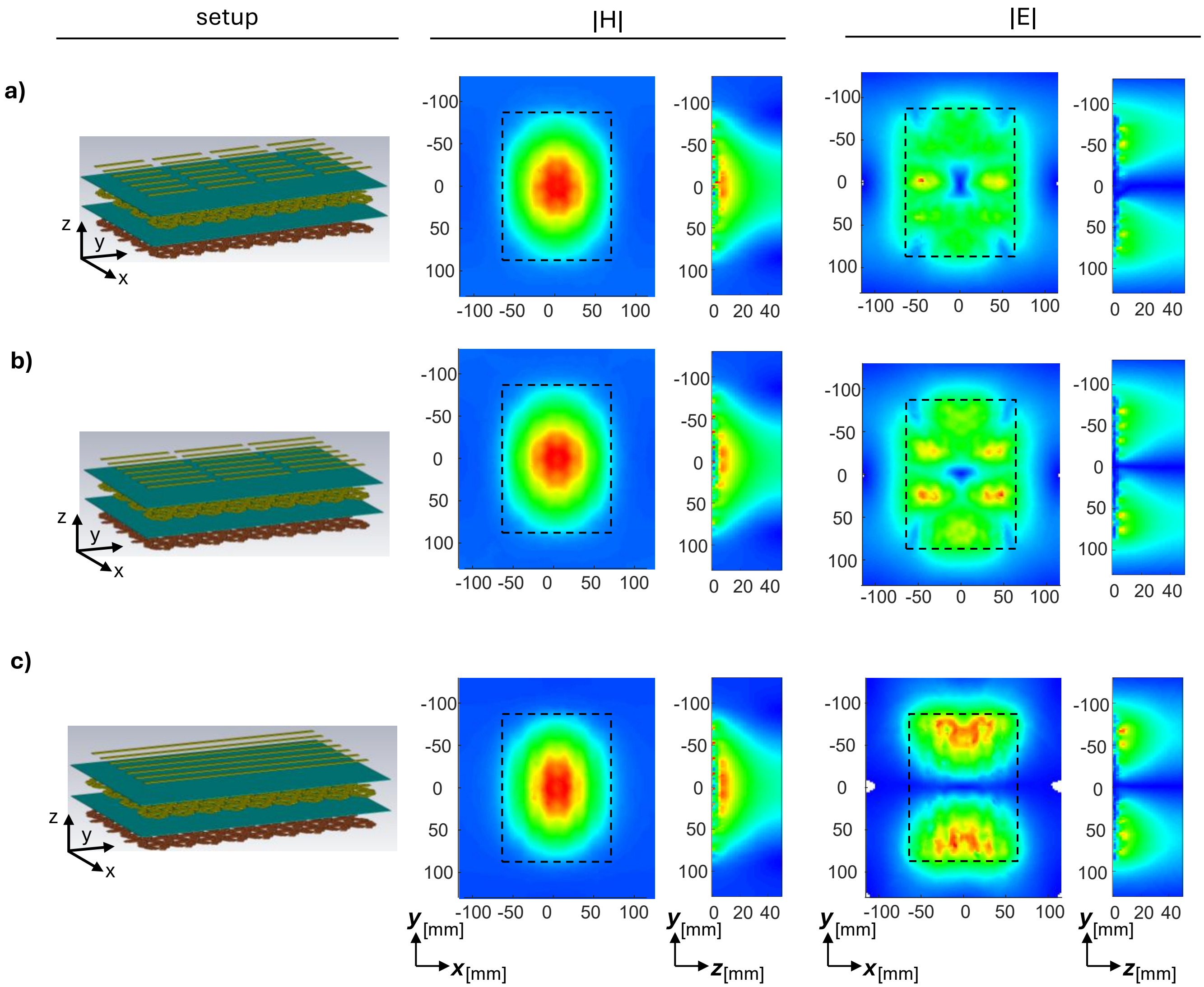

Figure S4. Resonant-mode magnetic and electric field distributions of hybrid artificial materials combining an artificial dielectric with an array of copper strips. (A) Artificial material with a 4 × 7 strip array. (B) Artificial material with a 3 × 7 strip array. (C) Artificial material with a 1 × 7 strip array. From left to right: structure schematics; |H| field maps in a plane parallel to the structure located 10 mm from its center and in a plane perpendicular to the structure; and |E| field maps in a plane parallel to the structure located 10 mm from its center and in a plane perpendicular to the structure. The black dashed outlines indicate the dimensions of the structures. All three configurations exhibit similar |H|-field distributions. The |E|-field distribution differs in (C), reflecting the more pronounced electric-dipole behavior of the single-strip-row configuration. In this case, the electric field is minimized near the center of the structure and maximized at the strip ends, consistent with the characteristic field distribution of electric dipoles.

S3. Phantom simulations and imaging

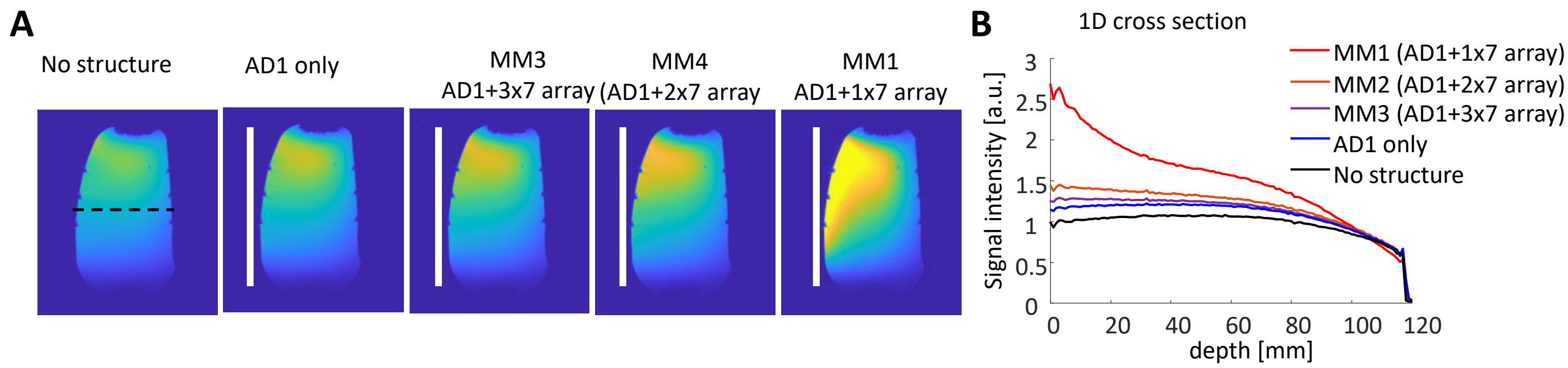

Figure S5. Phantom imaging at 7T using modular metamaterial configurations, including AD1 alone, MM3, MM2, and MM1. A) Magnitude images for each setup, B) 1D signal profile along the line indicated in the first image. The white box overlay shows the location of the metamaterial. The maximal intensity increase in the central line was 1.2, 1.3, 1.5,2.8 for AD1, MM3, MM2, and MM1, respectively. Assuming GRE scan intensity increases approximately with B_1^2, the maximal B_1^+ enhancement observed was 10%, 14%, 22% and 70%, respectively.

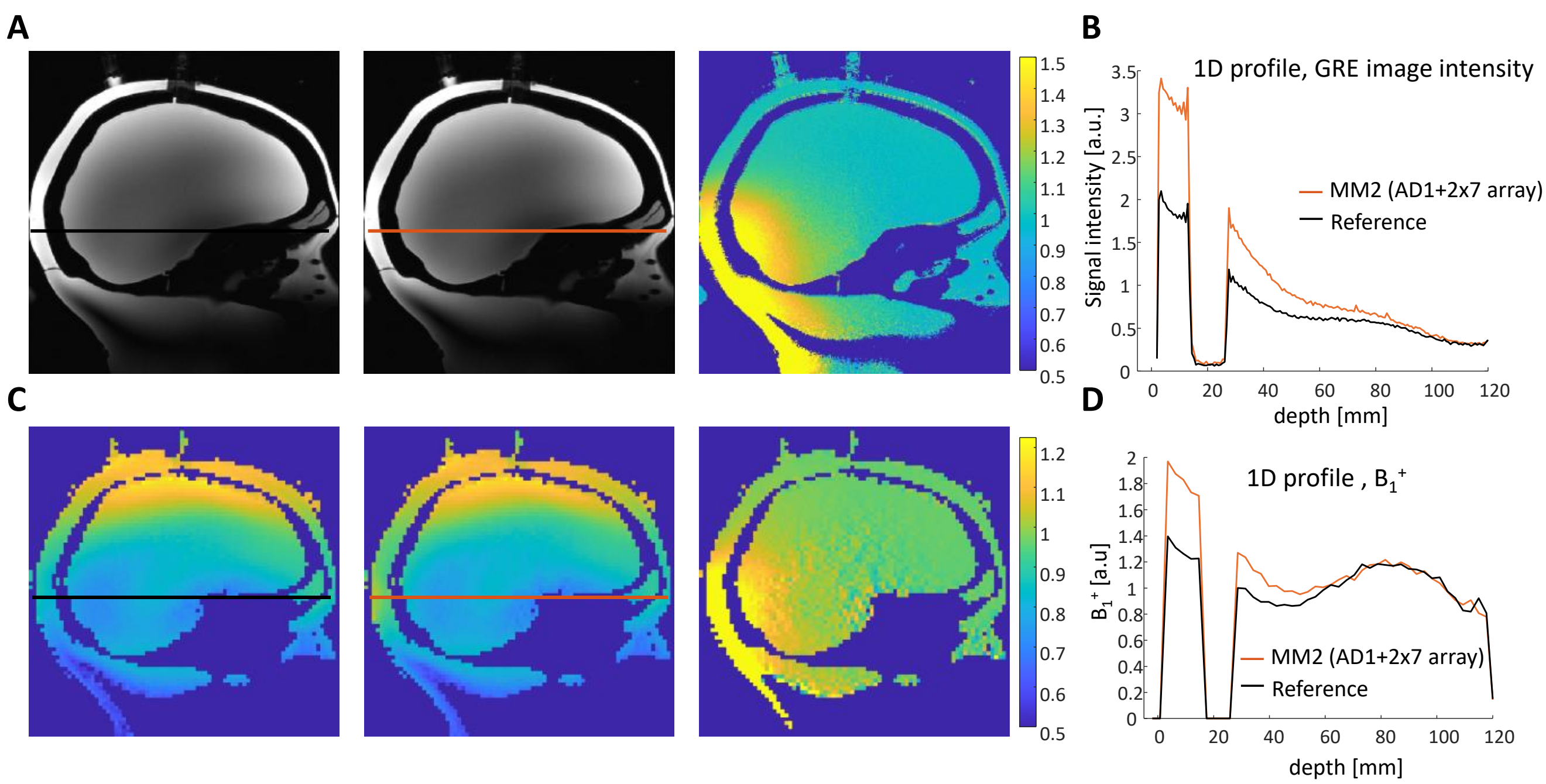

Figure S6. 3D head-shaped phantom imaging at 7T with the MM2 metamaterial configuration. A, B) MRI magnitude images acquired without and with the metamaterial, respectively. C, D) Corresponding B_1^+ maps without and with the metamaterial. The maximal intensity increase in the illustrated line (B) was 1.65 and maximal B_1^+ increase was 1.3. Ratio maps of the magnitude and B_1^+ images are shown in panels A and B, respectively.

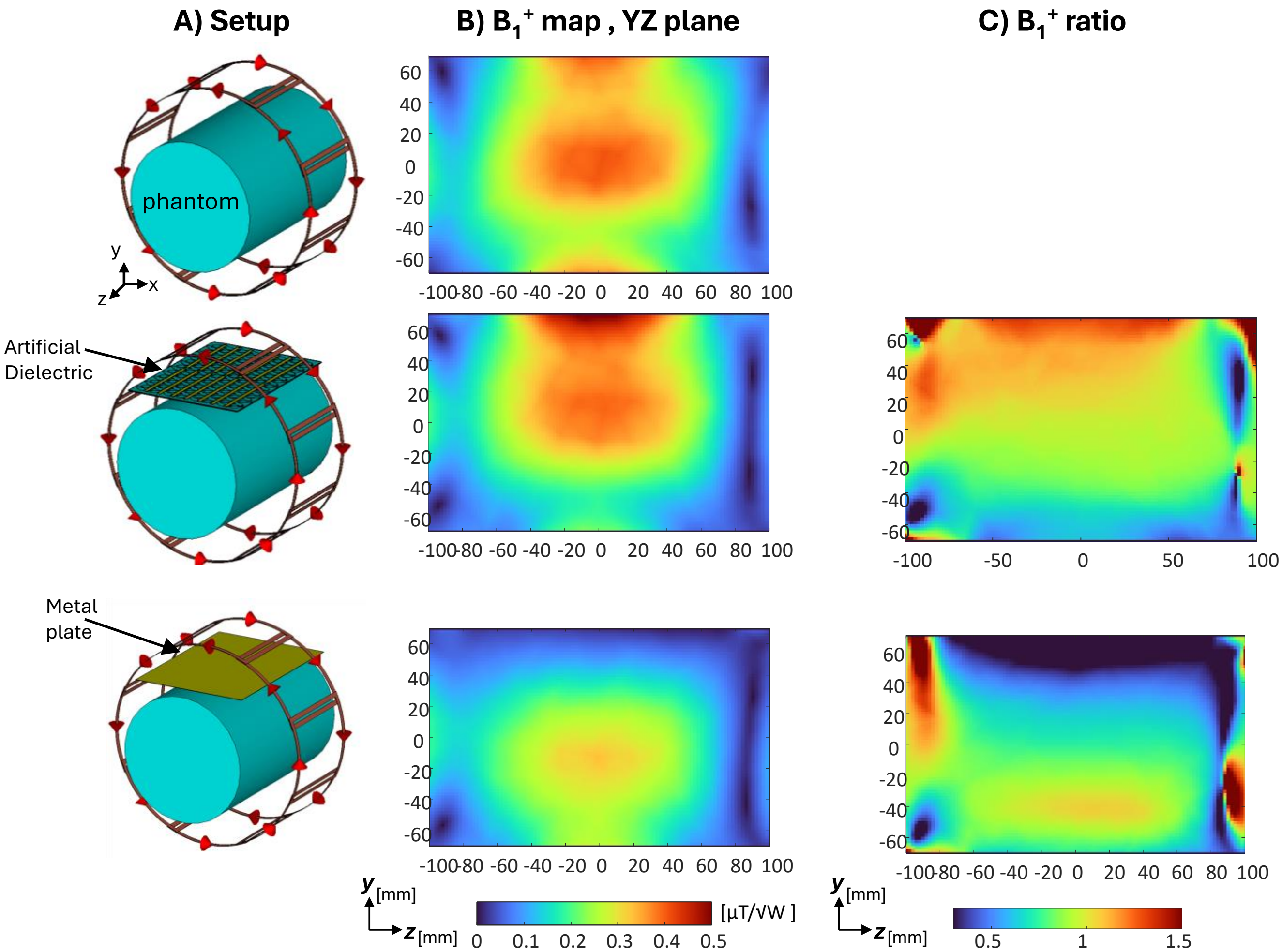

Figure S7. Simulations at 7 T under realistic coil-loading conditions using an 8-channel coil with receive dimensions comparable to those of a commercial Nova head coil and a head-sized cylindrical phantom. (A) Simulation setup. (B) B_1^+ field distributions in the YZ plane (perpendicular to the artificial dielectric). (C) B_1^+ ratio maps normalized to the reference case. Three configurations are shown: the reference setup, the artificial dielectric corresponding to Setup II in Fig. 2 with a 100-μm dielectric substrate, and a metal plate replacing the artificial dielectric. While the artificial dielectric increases the B_1^+ field, the metal plate results in signal reduction, confirming that the observed enhancement originates from the engineered electromagnetic response of the artificial dielectric rather than from the presence of conductive material alone.

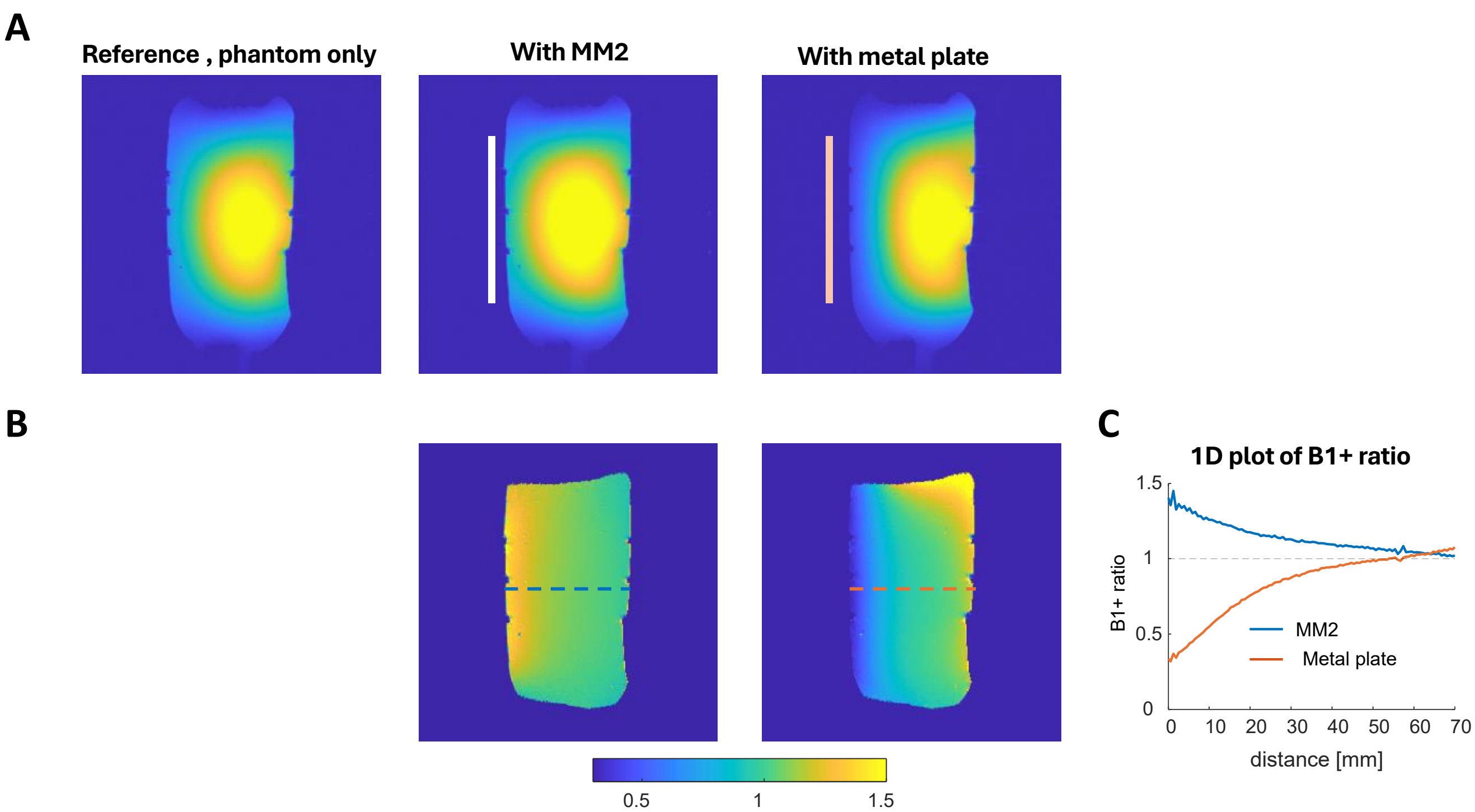

Figure S8. MRI comparison of the artificial material and a metal plate at 7 T. (A) GRE images acquired for the reference configuration, with the artificial material (MM2), and with a metal plate placed on top of the phantom. (B) Intensity ratio maps normalized to the reference image. (C) 1D intensity profiles through the center of the phantom. The artificial material (MM2) produced a 1.5-fold increase in signal intensity, whereas the metal plate reduced the signal to approximately 30% of the reference value.

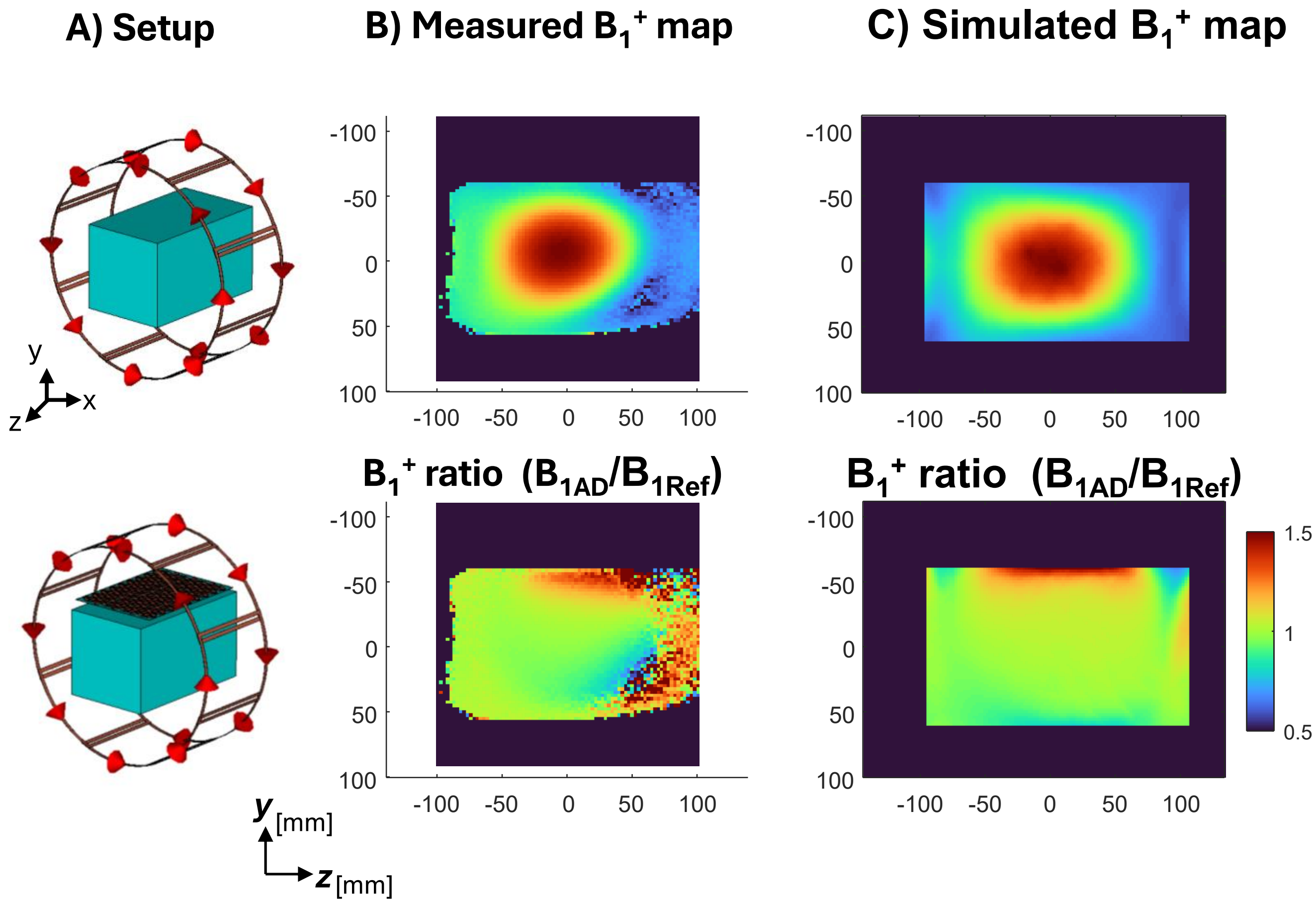

Figure S9. Comparison of measured and simulated B_1^+ maps at 7 T under realistic coil-loading conditions using an 8-channel coil with a transmit diameter of 30 cm, comparable to that of a commercial Nova head coil, and a phantom with brain-mimicking electrical properties (ε_r = 57, σ = 0.3 S/m, dimension 12cm × 12cm × 20cm). (A) Simulation setup. (B) Measured and (C) simulated B_1^+ field distributions in the YZ plane without the artificial dielectric, each normalized to its respective maximum, together with the corresponding B_1^+ ratio maps showing the enhancement produced by the artificial dielectric. Both measurement and simulation demonstrate a maximum B_1^+ enhancement of

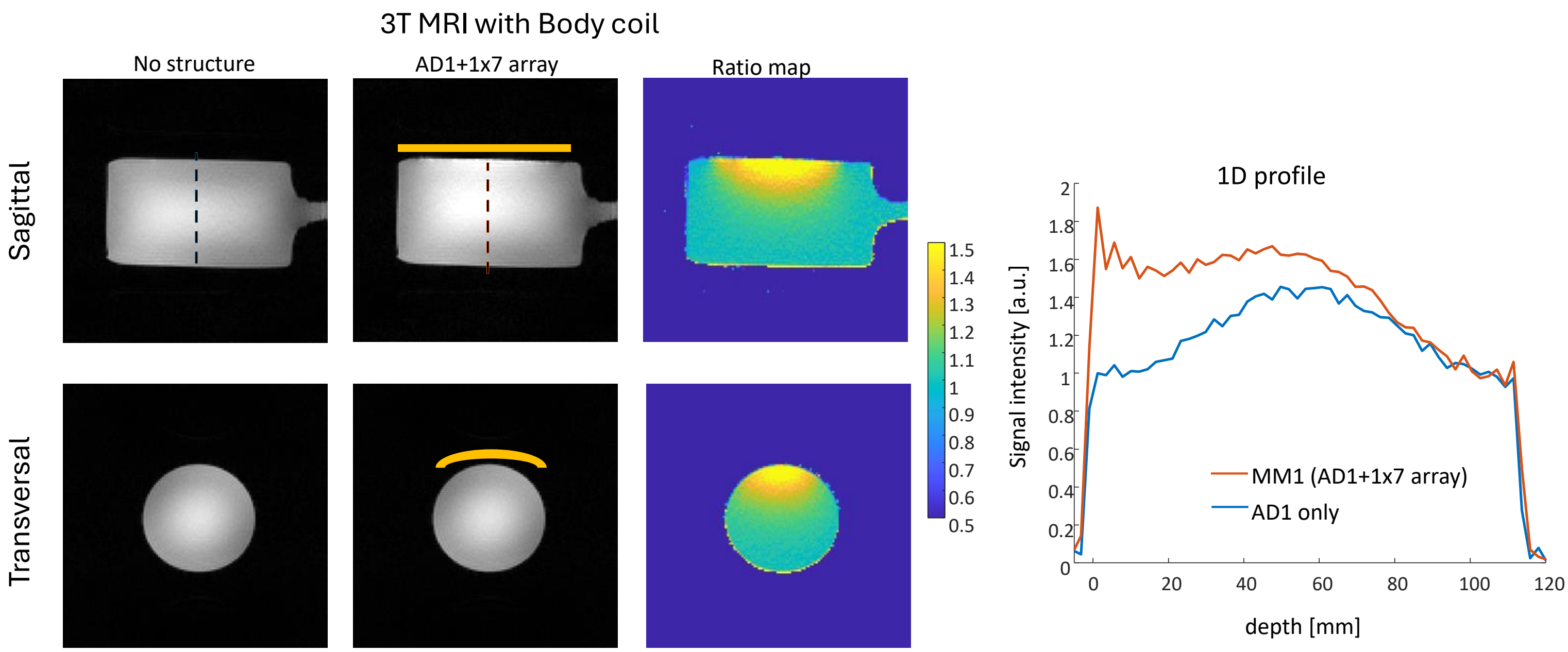

Figure S10. Phantom imaging at 3T with the MM1 configuration (AD1 + 1×7 dipole array). Sagittal and transverse images acquired without and with the metamaterial are shown, along with corresponding ratio maps. A 1D signal profile along the line indicated in the images is presented on the right.10

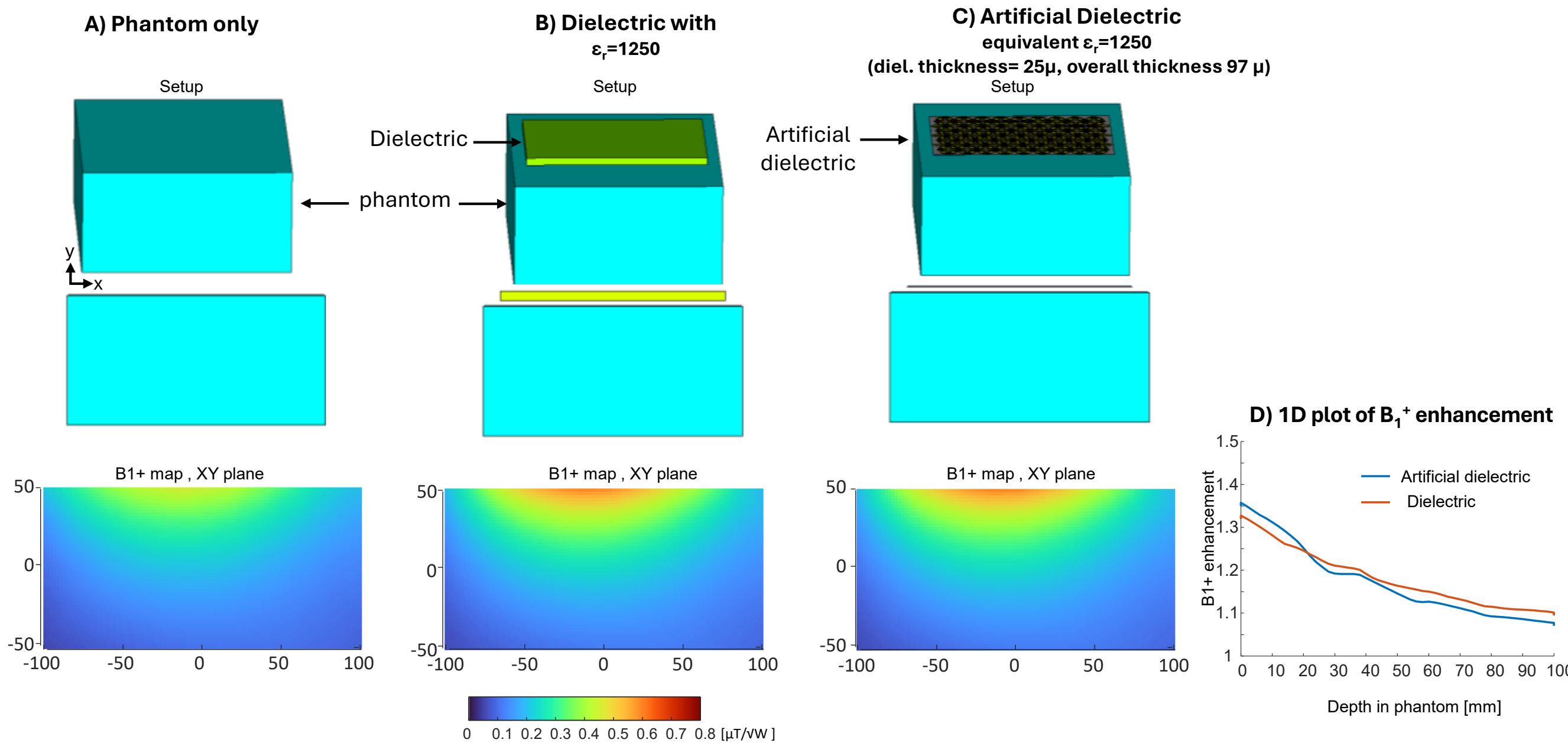

Figure S11. Simulations at 1.5 T comparing an artificial dielectric with a conventional dielectric. (A) Reference configuration consisting of the RF coil and phantom. (B) Conventional dielectric with $\varepsilon_r = 1250$. (C) Artificial dielectric with an effective $\varepsilon_r = 1250$, implemented using a 25-µm-thick dielectric substrate. From top to bottom: 3D view of the setup, corresponding 2D cross-sectional view, and B_1^+ field map. The left panel shows 1D profiles of the B_1^+ ratio normalized to the reference case. Both the conventional and artificial dielectric structures produce a similar enhancement, yielding approximately a 1.3-fold increase in the B_1^+ field.

S4. EM simulations with virtual human model

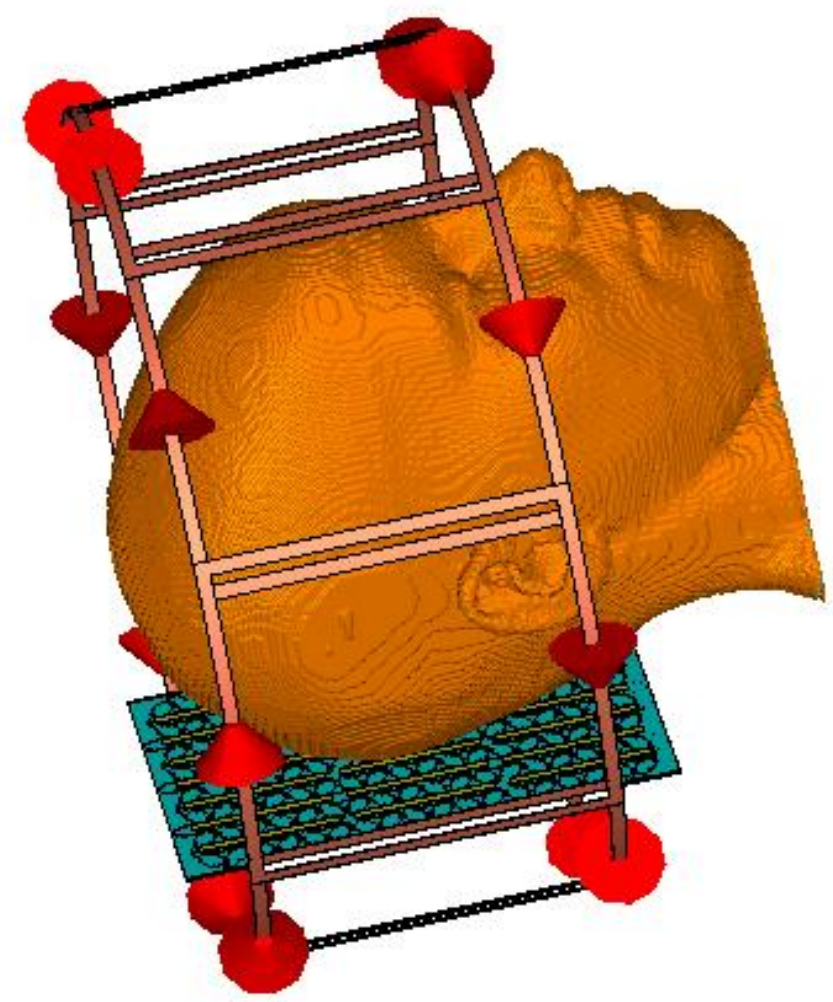

Figure S12. Schematic view of the setup used in EM simulations, including the 8-channels RF coil, the metamaterial positioned at the back of the head, and the human (Ella) head model.

S5. Photos of the metamaterial configurations

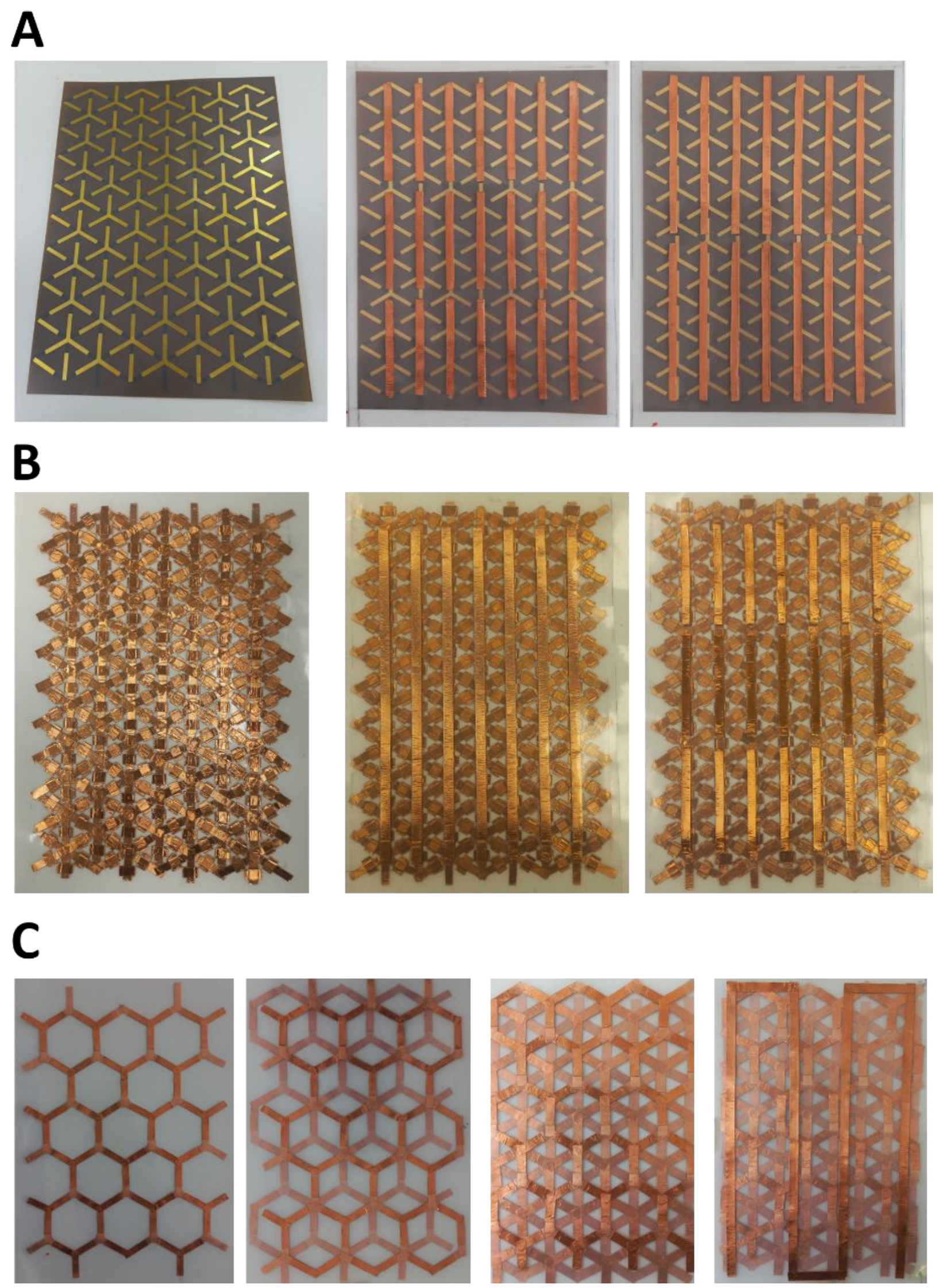

Figure S13. Photographs of the fabricated metamaterial configurations. A) Fig. 2 Setup II implemented using flexible PCB. From left to right: artificial dielectric only, with 3×7 dipole array, and with 2×7 dipole array. B) Fig. 2 Setup I low-cost implementation. From left to right: artificial dielectric only, with 1×7 dipole array and with 3×7 dipole array. C) Fig. 1C multilayer configuration. From left to right: single layer, dual-layer, triple-layer, and triple-layer with snake antenna.
